# Expanded character sampling inspired by a new Cretaceous conifer seed cone from California: importance of morphology in resolving relationships among the Cupressaceae

**DOI:** 10.1093/aob/mcaf099

**Published:** 2025-06-04

**Authors:** Kelly C Pfeiler, Alexander C Bippus, Ashley Ortiz, Ashley R Kammet, Ignacio H Escapa, Alexandru M F Tomescu

**Affiliations:** Department of Ecology and Evolutionary Biology, University of Kansas, Lawrence, KS 66045, USA; Biodiversity Institute, University of Kansas, Lawrence, KS 66045, USA; Department of Biological Sciences, California State Polytechnic University Humboldt, Arcata, CA 95521, USA; Department of Biological Sciences, California State Polytechnic University Humboldt, Arcata, CA 95521, USA; Department of Biological Sciences, California State Polytechnic University Humboldt, Arcata, CA 95521, USA; Consejo Nacional de Investigaciones Científicas y Técnicas and Museo Paleontológico Egidio Feruglio, Trelew, Chubut 9100, Argentina; Department of Biological Sciences, California State Polytechnic University Humboldt, Arcata, CA 95521, USA

**Keywords:** Anatomy, *Athrosequoia*, conifer, Cretaceous, Cupressaceae, fossil, phylogeny, seed cone

## Abstract

**Background and Aims:**

Cupressaceae are unique as a conifer family for their exceptionally high morphological diversity, particularly as expressed in seed cones. Their fossil record parallels this pattern and has yielded morphologies not represented in the modern flora. Describing diversity over the entire geological history of the family and using these data in a phylogenetic framework provides opportunities to explore evolutionary relationships among fossil and extant members to understand patterns of morphological evolution in the family.

**Methods:**

We describe a new cupressaceous fossil seed cone from the Early Cretaceous (Albian–Aptian boundary; 125 Mya) of California. We construct a novel set of discrete and continuous characters that sample exclusively seed cone morphology and anatomy to explore the evolutionary relationships of the new fossil among living and extinct Cupressaceae, with emphasis on the taxodiaceous grade.

**Key Results:**

The seed cone represents a new genus and species, *Athrosequoia walkeri* Pfeiler *et al.*, and the oldest member of the family having peltate ovuliferous complexes. *Athrosequoia walkeri* possesses a combination of characters encountered in several living taxodiaceous Cupressaceae subfamilies: Athrotaxidoideae, Sequoioideae and Taxodioideae. This novel combination of characters differentiates it from all previously known genera and consistently supports its stable phylogenetic placement among the taxodiaceous grade.

**Conclusions:**

This study documents additional morphological diversity of taxodiaceous Cupressaceae in the Early Cretaceous. The new character matrix highlights the power of seed cone characters in resolving phylogenetic relationships of fossil species, when combined with tree topology constraints based on results of molecular phylogenetics. Within this framework, additional taxon sampling, alongside denser sampling of morphological characters of living taxa, could provide excellent opportunities to understand both the phylogenetic position of extinct taxa and morphological evolution in the family.

## INTRODUCTION

The Cupressaceae *sensu lato* (i.e. including the former Taxodiaceae) are the most genus-rich family of conifers (31 genera). Six subfamilies are usually considered within the family: Cunninghamioideae (*Cunninghamia*; cunninghamioids hereafter), Taiwanioideae (*Taiwania*), Athrotaxidoideae (*Athrotaxis*; athrotaxidoids hereafter), Sequoioideae (*Sequoia*, *Sequoiadendron* and *Metasequoia*; sequoioids hereafter), Taxodioideae (*Taxodium*, *Cryptomeria* and *Glyptostrobus*; taxodioids hereafter), and Cupressoideae (including 22 genera; cupressoids hereafter). The Cupressaceae have an extensive fossil record comprising vegetative and reproductive structures (Stockey *et al.*, 2005). The earliest occurrence of seed cones unequivocally assignable to the family is the Early Jurassic *Austrohamia minuta*, described from the Pliensbachian/Toarcian of Patagonia (Escapa *et al.*, 2008; with dating constrained by *Cuneo et al.*, 2013), although other organs (e.g. anatomically preserved trunks) assigned to the family have older occurrences (Ruiz and Bodnar, 2019). Cupressaceous seed cones featuring ovuliferous complexes with some degree of peltate morphology are characteristic of the sequoioids and the athrotaxidoids and are also seen in some members of Cupressoideae (e.g. *Cupressus*, *Chamaecyparis* and *Hesperocyparis*). Fossils assignable to the cupressoids occur as early as the Cenomanian (Stockey *et al.*, 2005). Athrotaxidoid Cupressaceae are known from rocks as old as the Aptian–early Albian in the USA and Canada (Miller and LaPasha, 1983), China (Dong *et al.*, 2014 ) and Argentina (Del Fueyo *et al.*, 2008). They are represented by fossils placed in the genera *Athrotaxis* or *Athrotaxites* (Archangelsky, 1963; Miller and LaPasha, 1983; Stockey *et al.*, 2005 ; Escapa *et al.*, 2016). The oldest sequoioid, *Krassilovidendron fecundum* reported from Albian–Cenomanian strata in Russia (Sokolova *et al.*, 2017), possesses typical peltate ovuliferous complexes.

Relationships within the Cupressaceae have been explored in several studies in recent years. Most analyses that used only molecular data, only morphological data or combined the two place the sequoioids as sister to a clade that includes taxodioids and cupressoids (Gadek *et al.*, 2000; Kusumi *et al.*, 2000; Quinn *et al.*, 2002; Schulz and Stützel, 2007; Leslie *et al.*, 2012; Mao *et al.*, 2012). These analyses place *Athrotaxis* as sister to the clade including sequoioids, taxodioids and cupressoids. However, a different analysis based on molecular data (Yang *et al.*, 2012) places the sequoioids as sister to a clade including *Athrotaxis*, taxodioids and cupressoids. These results point to an unresolved polytomy between *Athrotaxis*, sequoioids and the clade that includes taxodioids and cupressoids, as shown in the analysis by Spencer *et al.* (2015) .

Several studies have suggested that seed cones might provide the most valuable information for exploring phylogenetic relationships among extant and extinct conifer taxa, and many phylogenetic analyses and taxonomic decisions in conifers are based on matrices that include seed cone features (Rothwell *et al.*, 2011; Leslie, 2011; Spencer *et al.*, 2015; Gleiser *et al.*, 2019). However, the studies on Cupressaceae that have included morphological data (Ohsawa, 1997; Gadek *et al.*, 2000; Quinn *et al.*, 2002; Farjon, 2005; Schulz and Stützel, 2007; Escapa *et al.*, 2008; Rothwell *et al.*, 2011; Mao *et al.*, 2012; Yang *et al.*, 2012; Shi *et al.*, 2014; Herrera *et al.*, 2017; Dong *et al.*, 2018; Atkinson *et al.*, 2021) have sampled relatively few seed cone characters; a maximum of 25 characters are used by Farjon (2005) and subsequently by Escapa *et al.* (2008), Rothwell *et al.* (2011) and Shi *et al.* (2014).

Here, we describe an anatomically preserved cupressaceous seed cone from deposits at the Barremian–Aptian (125 Mya) boundary in northern California. The cone, which shares structural features with those of sequoioid and athrotaxidoid cupressaceae, is older than the oldest known representatives of either of these two groups. Using phylogenetic analyses, we explore the role of detailed seed cone anatomical data in estimating phylogenetic relationships, with an emphasis on athrotaxidoids, sequoioids and taxodioids. Our analyses use a new set of discrete and continuous characters that we developed to sample aspects of seed cone morphology and anatomy not considered in previous phylogenetic studies. We also use these data to address the placement of the new fossil in the broader context of relationships between living and extinct Cupressaceae, as reflected by seed cone morphology. Throughout these analyses, we experiment with topological constraints to gain insights into the importance of morphological and anatomical seed cone features for resolving relationships within a conifer clade that has a deep fossil history and that encompasses more seed cone variability than any other conifer group.

## MATERIALS AND METHODS

### Geology

The Budden Canyon Formation is exposed on the southern flank of the Klamath Mountains, in northern California. The unit consists of a succession of clastic marine strata dominantly composed of mudstone that includes coarser-grained sequences of sandstone and conglomerate. Plant-fossiliferous near-shore marine deposits occur at several levels in the unit, with the richest plant fossil assemblages concentrated in the Hauterivian–Aptian interval (Murphy, 1956; Murphy *et al.*, 1969; Stockey and Smith, 2000; Burnett *et al.*, 2016). The plant material is preserved as carbonaceous compressions or permineralized in carbonate concretions along with marine invertebrates. The material presented here was collected from fossiliferous layers exposed in the banks of North Fork Cottonwood Creek, 1.5 km southeast of Ono, CA, USA (40°27′51″N, 122°36′32″W). These layers represent the upper section of the Lower Chickabally Member of the Budden Canyon Formation, close to its contact with the overlying Huling Member. This stratigraphic position places the fossil assemblage close to the Barremian–Aptian boundary, ∼125 Mya (see detailed discussion by Burnett *et al.*, 2016).

### Fossil material

The single isolated seed cone described here is permineralized by calcium carbonate and was studied using the cellulose acetate peel technique (Joy *et al.*, 1956). Two hundred peels document half of the cone in a series of oblique longitudinal sections; the other half of the cone was lost in the process of cutting the rock slab. Slides were prepared using Eukitt, a xylene-soluble mounting medium (O. Kindler Gmbh, Freiburg, Germany). Micrographs were taken using a Nikon Coolpix E8800 digital camera mounted on a Nikon Eclipse E400 compound microscope (Nikon, Minato, Tokyo, Japan). Images were processed using Photoshop (Adobe, San Jose, CA, USA). The three-dimensional rendering of the cone was produced using Amira (Thermo Fisher Scientific, Waltham, MA, USA and Konrad-Zuse-Zentrum für Informationstechnik, Berlin, Germany). All specimens and preparations are housed in the California State Polytechnic University Humboldt Paleobotanical Herbarium (HPH), Arcata, CA, USA.

### Phylogenetic study

#### Character construction and scoring

A set of 57 morphological seed cone characters was constructed and used to explore the importance of morphological and anatomical features in evaluating relationships within the Cupressaceae, with a focus on subfamilies Athrotaxidoideae, Sequoioideae and Taxodioideae (Supplementary Data Note). Our character construction approach was directed by several principles: (1) avoiding the inclusion of *ad hoc* hypotheses of homology that are implicit in the traditional conifer seed cone terminology [e.g. bract-scale complex, hereafter referred to as ovuliferous complex; see Escapa *et al.* (2016) and citations therein]; (2) parsing the complexity of seed cone morphology and anatomy into as many characters as possible; (3) constructing characters that handle ‘tail colour’-type characters as recommended by Hawkins *et al.* (1997) and Hawkins (2000); and (4) evaluating as continuous characters those features that (a) vary on a continuous scale and (b) describe the proportionality of cone structures, using ratios rather than absolute values.

Of the total of 40 discrete characters, 18 describe morphological features and 22 anatomical features (Supplementary Data Note). Among the continuous characters, ten describe the morphology and seven the anatomy of cone features. A variety of aspects of the morphology of ovuliferous complexes as seen in both adaxial and lateral views are captured by a combination of discrete and continuous characters. Such characters include the orientation of the distal region of the ovuliferous complex (exposed at the cone surface) with respect to its subtending portions; presence/absence of terminal lobes; distinctiveness of a basal ‘stalk’ portion; in addition to several continuous measures of ovuliferous complex external geometry. Internally, another subset of characters maps the vasculature of the ovuliferous complex by describing the relative position, geometry and orientation of the different components of the vascular bundle system. Differences between genera in the proportion of vascular tissue in the ovuliferous complex are captured in continuous characters based on cross-sectional surface areas. Other characters account for the anatomy of individual vascular bundles and presence of transfusion tissue. The system of resin canals is described in a similar way to the vasculature by discrete and continuous characters. To avoid emphasizing differences in the absolute sizes of structures, which might not bear a phylogenetic signal and can be misleading in a comparative framework, the majority of continuous characters describe differences in the proportionality of cone structures, using ratios of variables rather than absolute values (e.g. character 5: Basal cone axis diameter:narrowest size of ovuliferous complex stalk/base).

Characters were scored from the published literature and by examining material in our own Cupressaceae cone collection at California State Polytechnic University Humboldt (Supplementary Data Note; Supplementary Data Sheets S1–S3); scores were recorded using Mesquite v.3.2 software (Maddison and Maddison, 2009). Measurements of continuous characters were performed using ImageJ (National Institutes of Health, Bethesda, MD, USA). For consistency of character scoring, scores for each taxon were based on the most developed ovuliferous complexes, i.e. those located in the mid-section of the cone, away from the base and apex. When provided in the literature, the variation of a character among specimens within a species was scored as a range. For continuous characters where such ranges of natural variation have not been reported, we created an artificial range for each character by bracketing the single value available by ±10 %, to avoid artificially emphasizing differences between taxa for which ranges of variation are not available. The complete range of each character was standardized as the equivalent to one step of a discrete character change, as detailed by Bippus *et al.* (2018). The 17 continuously varying features were treated as continuous characters, using the methods proposed by Goloboff *et al.* (2006).

The scored matrix has 19.6% missing data. Discrete character scorings are reported in Supplementary Data Sheet S1 and the raw data for continuous characters in Supplementary Data Sheet S2. Supplementary Data Sheet S3 is the phylogenetic matrix, including discrete and continuous data. Tree files are provided in Supplementary Data Sheet S4.

#### Taxon sampling

Our analyses include nine taxodiaceous living genera of the Cupressaceae (excluding *Taiwania*); eight fossil species that preserve sufficient anatomical detail, possess peltate ovuliferous complexes and are putatively related to living *Athrotaxis*, sequoioids or taxodioids; *Cunninghamiostrobus hueberi* as a close relative of the outgroup, *Cunninghamia*; and *Archicupressus nihongii*, as a fossil cupressoid representative (Supplementary Data Note). For extant genera that have multiple species, we scored the following species: *Hesperocyparis macrocarpa*, *Cunninghamia lanceolata*, *Athrotaxis laxifolia* and *Taxodium distichum*. We chose these species because we had access to their cones at varying stages of maturity and were able to score characters from personal observations, to increase consistency in scoring among taxa. We did not include *Taiwania* in part because of a lack of cone material in our collection for detailed direct observations and because our focus was on cones with peltate to sub-peltate ovuliferous complexes, unlike the foliate ovuliferous complexes of *Taiwania*. Because here we are trying to obtain more resolution for a specific subset of relationships within the Cupressaceae instead of addressing the overall pattern of phylogeny in the family, we have used extant *Cunninghamia* as the outgroup, because this genus is consistently recovered as the sister to the rest of the family in most of the recent phylogenies (Gadek *et al.*, 2000; Quinn *et al.*, 2002; Schulz and Stützel, 2007; Leslie *et al.*, 2012; Mao *et al.*, 2012; Yang *et al.*, 2012; Shi *et al.*, 2014).

#### Phylogenetic analyses

Phylogenetic searches were conducted in the program TNT v.1.5 (Goloboff and Catalano, 2016) using equally weighted parsimony as the optimality criterion. The parsimony analyses were initiated using the command *xmult = hits10*. Using this command, the analysis starts from 50 random addition sequences, which are refined by tree bisection–reconnection. The resulting trees are then submitted to a combination of ratchet (default settings), tree drifting (default settings) and sectorial searches (default settings).

#### Analyses and questions addressed

In our experimental approach aimed at exploring the role of the new morphological characters in improving the definition of phylogenetic relationships for fossil species, we used several sets of analyses that sampled different combinations of taxa, characters and constraints on relationships (Table 1). A first set of analyses sampled extant genera plus the new fossil genus, *Athrosequoia*, only. Under this taxon sampling, we conducted tree searches without constraining relationships, based on discrete plus continuous characters (Analysis 1.A.1) and based on discrete characters only (Analysis 1.A.2), in addition to tree searches where relationships between living genera were constrained based on the results of Leslie *et al.* (2012), using discrete plus continuous characters (Analysis 1.B.1). This first set of analyses addressed: (1) the position of *Athrosequoia* among living cupressaceous genera, as resolved by morphological seed cone characters; (2) the effect of topological constraints in terms of topological congruence between our set of seed cone morphological characters and the mostly DNA-supported currently accepted topology for Cupressaceae; and (3) the phylogenetic role of discrete and continuous morphological seed cone characters.

**Table 1.** Summary of phylogenetic analyses of living taxa and *Athrosequoia*.

Analysis	1.A.1	1.A.2	1.B.1
Character sampling	Discrete + continuous	Discrete only	Discrete + continuous
Constrained analysis	—	—	+
Tree length (number of MPTs)	77.2 (1 MPT)	64 (3 MPTs)	82.04 (1 MPT)
Sister taxon of *Athrosequoia*	(Sequoioids + (*Hesperocyparis*, taxodioids))	(Sequoioids + *Hesperocyparis* + (taxodioids))	(Sequoioids (*Hesperocyparis*, taxodioids))
Sister taxon of *Athrotaxis*	(*Athrosequoia* (sequoioids + (*Hesperocyparis*, taxodioids)))	(*Athrosequoia* (sequoioids + *Hesperocyparis* + (taxodioids)))	(*Athrosequoia* (sequoioids (*Hesperocyparis*, taxodioids)))
Sequoioids	Paraphyletic	Part of polytomy with *Hesperocyparis* and taxodioids	Monophyletic
Sister taxon of sequoioid clade	—	—	(*Hesperocyparis*, taxodioids)
Taxodioids	Monophyletic	Monophyletic	Monophyletic
Sister taxon of taxodioid clade	*Hesperocyparis*	? (Polytomy with *Hesperocyparis* and sequoioids)	*Hesperocyparis*
Sister taxon to *Hesperocyparis*	Taxodioids	? (Polytomy with sequoioids and taxodioid clade)	Taxodioids

Abbreviation: MPT = most parsimonious tree. Question marks denote unresolved relationships.

A second set of analyses sampled all extant and fossil taxa. Under this taxon sampling, we ran tree searches without constraining relationships, based on discrete plus continuous characters (Analysis 2.A.1) and based on discrete characters only (Analysis 2.A.2), in addition to tree searches with relationships between living genera constrained based on the results of Leslie *et al.* (2012), using discrete plus continuous characters (Analysis 2.B.1). The second set of analyses addressed: (1) the position of *Athrosequoia* among living and fossil cupressaceous taxa, as resolved by morphological seed cone characters; (2) the effect of topological constraints in terms of topological congruence between our set of seed cone morphological characters and the mostly DNA-supported currently accepted topology for Cupressaceae; and (3) the phylogenetic effects of discrete and continuous morphological seed cone characters, when many fossil species are included.

## RESULTS

### Systematics

Order— Cupressales Link

Family—Cupressaceae Bartlett

Genus—*Athrosequoia* Pfeiler, Ortiz et Tomescu gen. nov.

#### Generic diagnosis

Woody seed cone containing many helically arranged peltate ovuliferous complexes. Cone axis containing large amounts of secondary xylem without growth rings and composed of tracheids with circular bordered pits and taxodioid-type crossfield pitting. Ovuliferous complexes with rhomboidal to pentagonal peltate heads bearing a horizontal groove on the distal surface. Vascular traces supplying the ovuliferous complexes diverge perpendicular to the cone axis and concentrate in the adaxial portion of the ovuliferous complex. Large resin canals present abaxial to the vascular tissues in the ovuliferous complexes. Cone subtended by several small scale-leaves with helical taxis.

### Type species *Athrosequoia walkeri* Pfeiler, Ortiz & Tomescu sp. nov.

#### Etymology

The generic name *Athrosequoia* refers to the morphological features that the fossil shares with extant members of the Athrotaxidoideae, Sequoioideae and Taxodioideae.

### Species *Athrosequoia walkeri* Pfeiler, Ortiz & Tomescu sp. nov.

#### Specific diagnosis

Seed cone ovoid in shape, 9 mm long, 6 mm in diameter, with 22–28 helically arranged peltate ovuliferous complexes. Cone axis 6.5 mm long, 1 mm wide. Narrow parenchymatous pith 120–170 µm in diameter runs the length of the cone. Secondary xylem of cone axis forming continuous cylinder, ∼300 µm in radius, with narrow tracheids with uniseriate circular bordered pits. Xylem rays four to six cells high, uniseriate. Ovuliferous complexes ≤2 mm in length, with peltate heads 2.6 mm wide and 2 mm tall. Three longitudinal resin canals positioned mid-way between the adaxial and the abaxial side of ovuliferous complex. Largest resin canal runs most of the length of the ovuliferous complex. Two smaller lateral canals parallel to the central canal. Xylem trace of ovuliferous complex 190–340 µm in diameter, with adaxially eccentric narrow pith. Scale-like leaves at the base of the cone 0.7 mm wide, 0.5 mm thick and 1 mm long. Leaf apices acute and upturned, leaves containing large resin canal, 170 µm in diameter. Leaf trace is adaxially adjacent to the resin canal and flanked by transfusion tissue.

#### Holotype

Seed cone in accession HPH 223 C (Cal Poly Humboldt Paleobotanical Herbarium).

#### Type locality

Right bank of North Fork Cottonwood Creek, 1.5 km southeast of Ono, CA, USA; 40° 27′ 51″ N; 122° 36′ 32″ W.

#### Stratigraphic position and age

Upper section of the Lower Chickabally Mudstone Member; Barremian–Aptian boundary, Early Cretaceous, ∼125 Mya.

#### Etymology

The specific epithet honours the work of Professor Dennis K. Walker, who established and enriched the living conifer collection at Humboldt State University (currently Cal Poly Humboldt); this collection provided much of the material used in this study to document seed cone anatomy in extant cupressaceous conifers, thus making possible the analysis that allowed for phylogenetic placement of the fossil cone.

#### Description

**Fig. 1.**
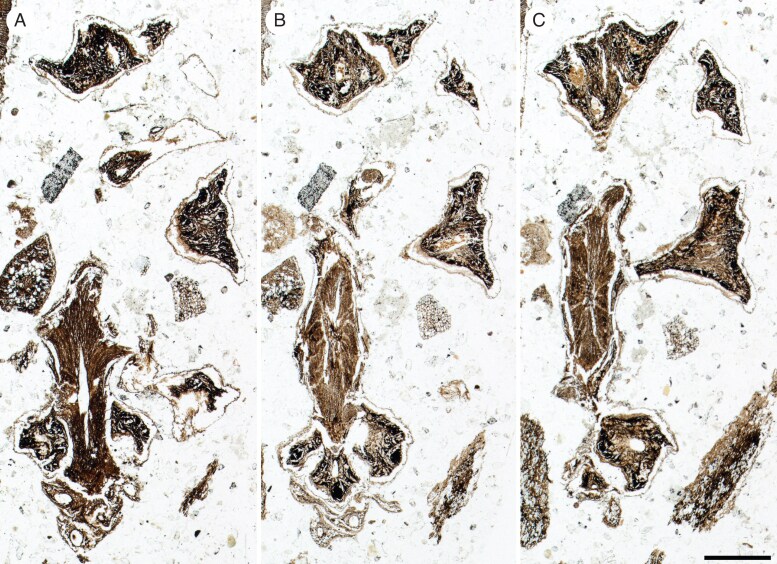
Overall morphology and anatomy of *Athrosequoia walkeri* gen. et sp. nov. in oblique longitudinal sections at selected sections. In all sections, note the cone axis with secondary xylem and a narrow pith and the small scale-like leaves subtending the cone. See also the thin parenchymatous layer of radially flattened cells that surrounds all parts of the cone. Scale bar: 1 mm. HPH223 Ctop 25a (A), 16a (B) and 7a (C).

**Fig. 2.**
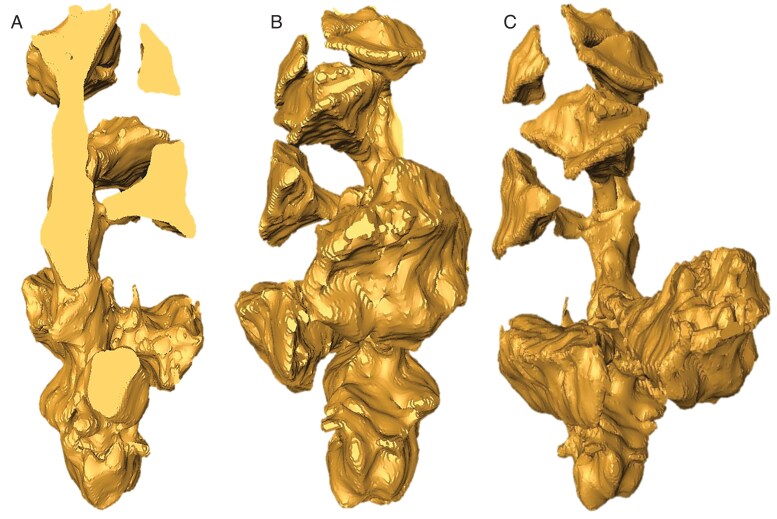
Three-dimensional rendering of the seed cone *Athrosequoia walkeri* gen. et sp. nov. (A–C) Three-dimensional rendering in different planes of view. Note ovuliferous complexes helically arranged with peltate heads rhomboidal to pentagonal in shape and horizontal grooves on distal surfaces.

The specimen represents a longitudinal half of a seed cone, consisting of the cone axis with attached ovuliferous complexes (Figs 1 and 2). The cone is ovoid in shape, 9 mm long, 6 mm in diameter, with a total of 22–28 helically arranged peltate ovuliferous complexes. This total number of ovuliferous complexes is an estimate based on the number of ovuliferous complexes (and ovuliferous complex bases) counted on the half-cone, multiplied by two. Although the cone exhibits features inconsistent with a senescent stage (see Discussion), it appears to be dehisced, and no seeds or ovules are preserved inside (Fig. 1). All cone parts are surrounded by an incompletely preserved, thin (95–145 µm) parenchymatous layer composed of radially flattened cells (Figs 1 and 6D).

**Fig. 3.**
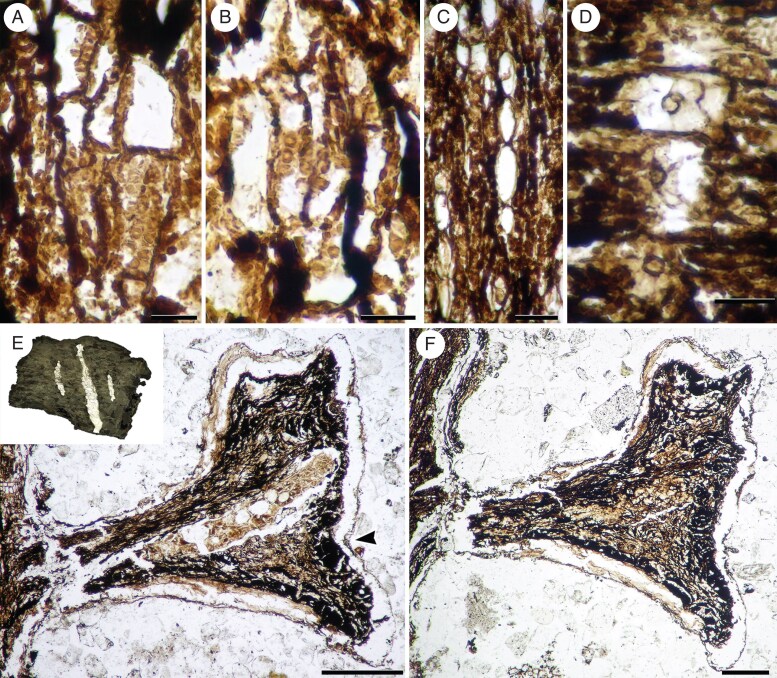
Anatomical details of xylem and ovuliferous complexes in *Athrosequoia walkeri* gen. et sp. nov. (A and B) Tracheids of secondary xylem in the cone axis, with uniseriate circular bordered pits. Scale bar: 20 µm. HPH223 Ctop 132a (A) and 134a (B). (C) Uniseriate xylem rays, four to six cells high, in the secondary xylem of the cone axis. Scale bar: 30 µm. HPH223 Ctop 7a. (D) Taxodioid-type crossfield pitting of tracheids. Scale bar: 20 µm. HPH223 Ctop 7a. (E and F) Longitudinal sections through an ovuliferous complex showing peltate morphology and horizontal groove (arrowhead) on the distal surface of the peltate head. (E) Note large resin canal that runs through most of the length of the ovuliferous complex as seen in longitudinal section. Inset represents a three-dimensional rendering of the three resin canals present in the ovuliferous complex; two smaller canals that run parallel to the central canal; note that a resin canal is not present at the very base of the ovuliferous complex. Scale bar: 500 µm. HPH223 Ctop 0a. (F) Scale bar: 300 µm. HPH223 Ctop 3a.

The cone axis is 6.5 mm long, 0.96 mm wide at its thickest diameter and is composed primarily of secondary xylem, with no observable growth rings (Fig. 1). An incompletely preserved narrow parenchymatous pith measuring 120–168 µm in diameter runs the length of the cone axis (Fig. 1). Surrounding the pith, secondary xylem forms a continuous cylinder, ∼310 µm in radius, consisting of narrow, radially aligned tracheids with uniseriate circular bordered pits (Fig. 3A, B). Xylem rays are four to six cells high, formed of cells 24–36 µm high, 12–15 µm wide and 24–34 µm long (Fig. 3C). Crossfield pitting is of the taxodioid type (Baas *et al.*, 2004) (Fig. 3D). Tissues outside the vascular cylinder are incompletely preserved, and neither a vascular cambium nor secondary phloem can be distinguished within the extraxylary region.

**Fig. 4.**
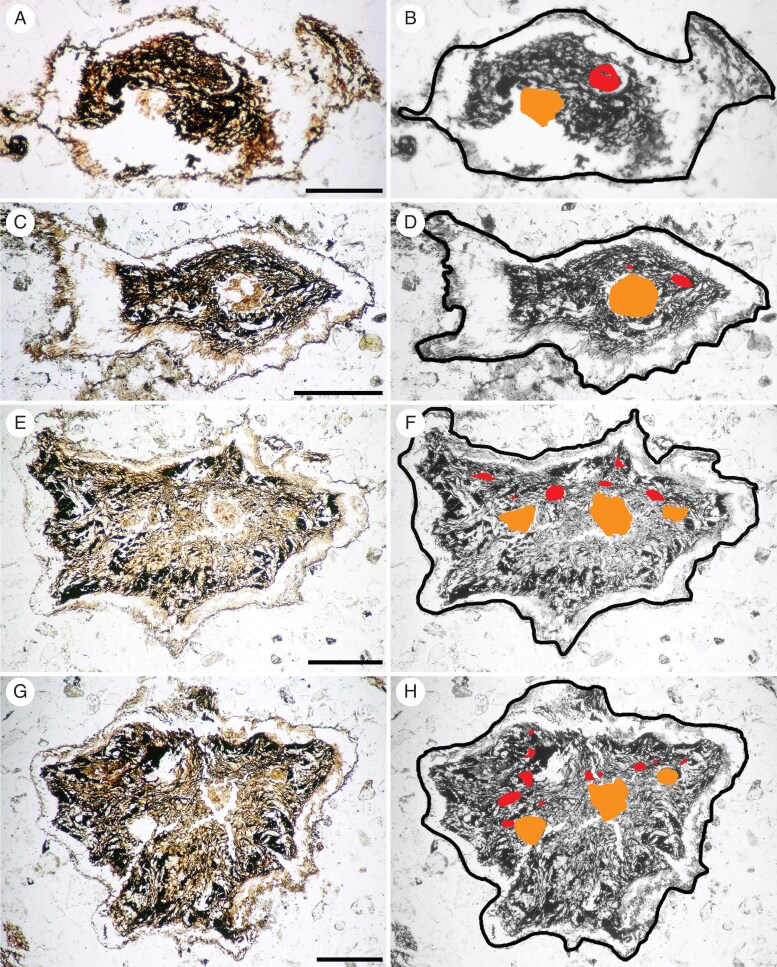
Anatomical details of ovuliferous complex *Athrosequoia walkeri* gen. et sp. nov. Selected serial sections from the base (top) to distal portions (bottom) of ovuliferous complex in cross-section; right panel highlighting ovuliferous complex outline (black), vascular tissues (red) and resin canals (orange). (A and B) Sections near the base of ovuliferous complex with adaxially eccentric xylem cylinder positioned above the resin canal. (C–H) Note vascular trace branching to form many minute vascular bundles. The distal regions of the ovuliferous complex contain many vascular bundles forming an oval figure in the adaxial half of the complex, above the three resin canals (G and H). (A and B) Scale bar: 50 µm. HPH223 Ctop 74a. (C and D) Scale bar: 50 µm. HPH223 Ctop 104a. (E and F) Scale bar: 500 µm. HPH223 Ctop 134a. (G and H) Scale bar: 500 µm. HPH223 Ctop 145a.

A volume rendering of the cone reveals the helical arrangement and the morphology of the ovuliferous complexes (hereafter referred to as OC; Fig. 2). Ovuliferous complexes feature thin stalks and peltate heads. The OC are ≤2 mm in total length, with stalks 960 µm in diameter at the base. The stalks abruptly broaden to form the peltate heads that are 2.6 mm wide and 2 mm tall. The distal surface of the peltate heads is rhomboidal to pentagonal in shape and has a horizontal groove (seen in both longitudinal sections and in the volume rendering; Figs 2 and 3E, F). A mucro (also referred to in some works as bract tip) is present in the middle of the horizontal groove. An OC cut in cross-section shows three resin canals positioned mid-way between the adaxial and the abaxial side (Fig. 4). The largest runs centrally for most the length of the OC and is 300 µm in diameter (Fig. 3E). Two lateral canals, 180 µm in diameter, run parallel to the central canal in the distal half of the OC and are not connected to the central canal (Fig. 3E).

**Fig. 5.**
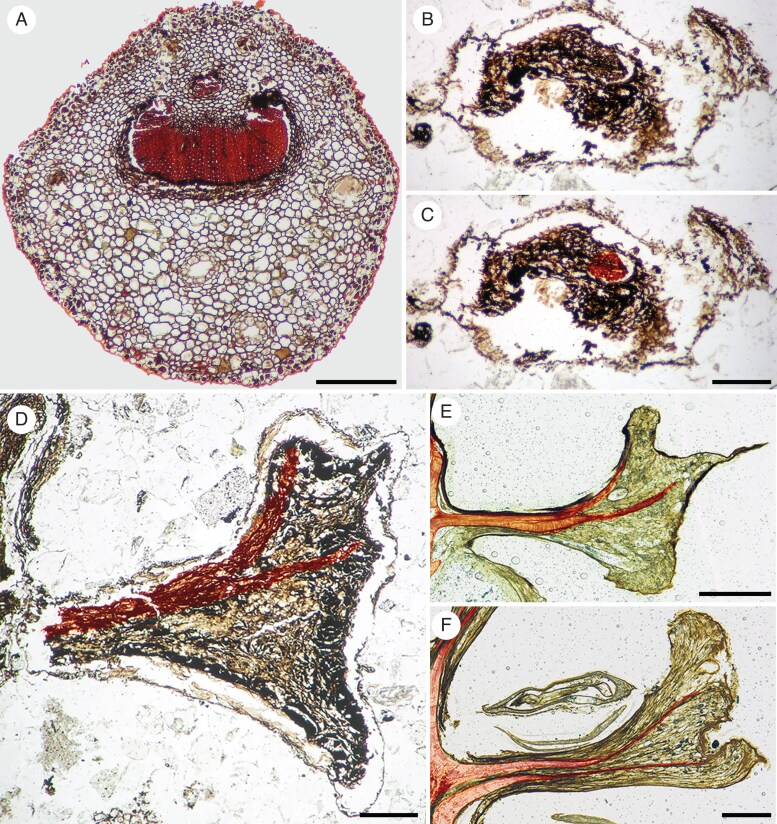
Comparisons of vascular trace anatomy of *Athrosequoia walkeri* gen. et sp. nov. and living members of Cupressaceae. (A) Adaxially eccentric pith of xylem cylinder of *Athrotaxis* located near the base of the ovuliferous complex. Scale bar: 200 µm. (B and C) *Athrosequoia* base of ovuliferous complex, with the pith of the xylem cylinder adaxially eccentric; highlighted in red in C. Scale bar: 200 µm. HPH223 Ctop 74a. (D–F) Vertical longitudinal section of the ovuliferous complex in *Athrosequoia* (D), *Athrotaxis* (E) and *Metasequoia* (F), showing trace of ovuliferous complex separating to form adaxial and abaxial components (highlighted in red). In *Athrosequoia* (D) and *Athrotaxis* (E), the vascular cylinder supplying the ovuliferous complex is situated in the upper half of the ovuliferous complex, and the abaxial component of the cylinder supplies the distal peltate head towards the mucro, whereas in *Metasequoia* (F) the cylinder is located medially in the ovuliferous complex stalk, and the abaxial component of the vascular cylinder supplies the peltate head below the horizontal groove. (D) Scale bar: 300 µm. HPH223 Ctop 3a. (E) Scale bar: 2 mm. (F) Scale bar: 1 mm.

**Fig. 6.**
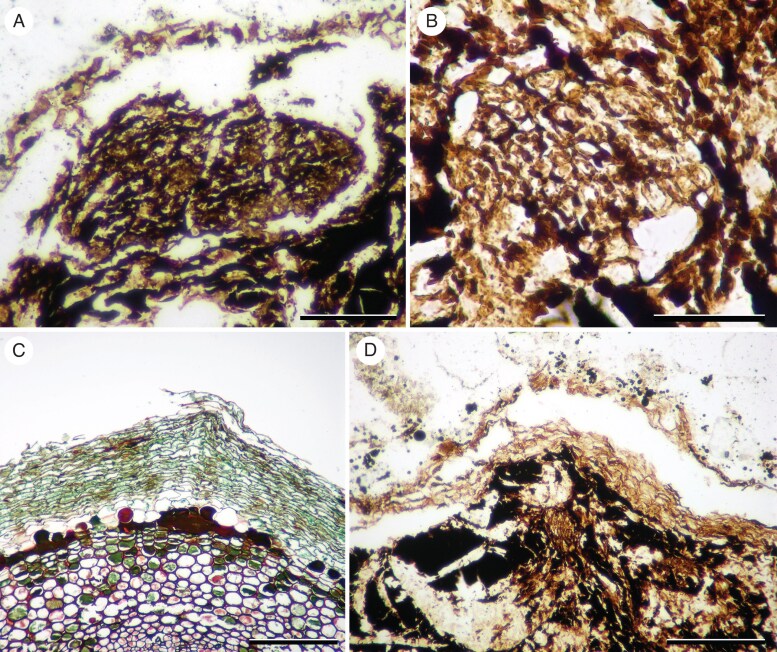
(A) Xylem cylinder supplying the basal region of the ovuliferous complexes of *Athrosequoia*. Scale bar: 100 µm. HPH223 Ctop 71a. (B) Vascular bundle in distal regions of the peltate head in *Athrosequoia* consisting of radially aligned tracheids. Scale bar: 50 µm. HPH223 Ctop 134a. (C) Parenchymatous tissue present in nearly mature yet still green cones of living *Sequoia*. Scale bar: 200 µm. (D) Parenchymatous layer surrounding *Athrosequoia* that is similar to the layer seen in *Sequoia* and supports the interpretation that *Athrosequoia* could have been mature yet still green at the time of fossilization. Scale bar: 200 µm. HPH223 Ctop 3a.

The xylem trace supplying the OC diverges perpendicularly from the cone axis as a cylinder, 192–336 µm in diameter, positioned adaxially with respect to the central resin canal (Figs 4A, B, 5B and6A). At the base of the OC, the pith of the OC trace is adaxially eccentric within the xylem cylinder, similar to that seen in *Athrotaxis* (Fig. 5A), and the xylem features a few uniseriate rays. In vertical longitudinal sections of the OC, the trace is seen splitting at the junction between stalk and peltate head to form adaxial and abaxial components (Fig. 5D). The adaxial component follows the upper OC surface, whereas the abaxial component continues straight towards the mucro, supplying the distal peltate head region. Based on its position close to the surface of the OC, the adaxial component probably includes bundles that supply the seeds, although direct evidence for this is not available, owing to incomplete preservation and the absence of seeds. Serial cross-sections of OCs indicate that the adaxial and abaxial components each branch further to form multiple minute, inconspicuous vascular bundles within the peltate head (Fig. 4). Although these minute bundles are difficult to trace across all the serial sections, owing to their size, in the peltate head they are located in the adaxial half of the OC, where they seem to form an oval figure consisting of ≥11 bundles (Fig. 4G, H). These bundles consist of radially aligned tracheids ≤17 µm in diameter (Fig. 6B). Around the vascular bundles and resin canals, the OC tissues are heavily sclerified (Fig. 4G).

**Fig. 7.**
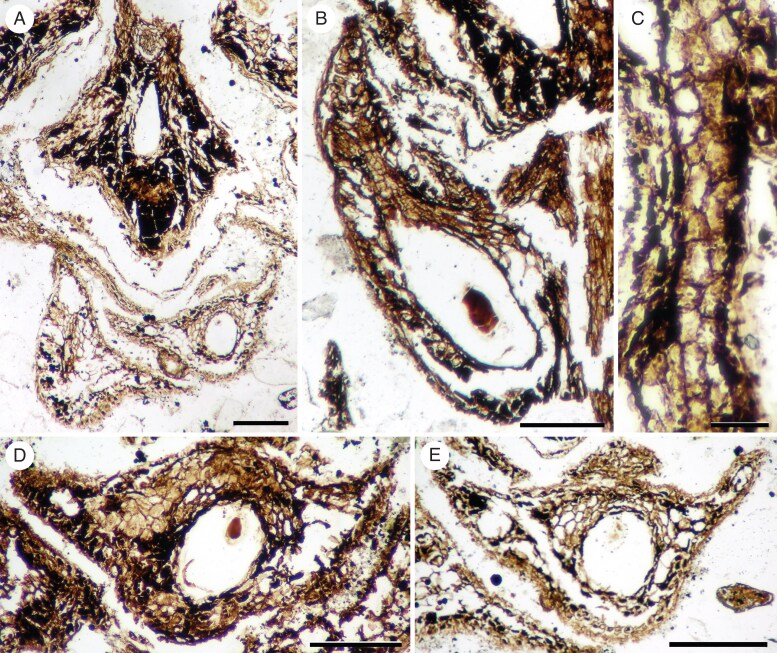
Anatomy of basal ovuliferous complex and scale-like leaves subtending seed cone of *Athrosequoia walkeri* gen. et sp. nov. (A) Basal ovuliferous complex in cross-section at top, with vascular bundle seen adaxially and a large central resin canal. Note also the scale like-leaves that subtend the cone. Scale bar: 200 µm. HPH223 Ctop 14a. (B) Longitudinal section of scale-like leaves, showing acute upturned apex and large central resin canal with vascular tissues positioned adaxially of canal. Scale bar: 200 µm. HPH223 Ctop 48a. (C) Detail of leaf trace in longitudinal section. Scale bar: 50 µm. HPH223 Ctop 54a. (D and E) Cross-section of leaves, rhomboidal to crescent-shaped, with large, flattened vein abaxially of resin canal flanked by transfusion tissue. Scale bar: 200 µm. HPH223 Ctop 61a (D) and 16a (E).

The cone is subtended by eight scale-like leaves that are attached to its peduncle. The leaves are rhomboidal or crescent shaped in cross-section, ∼0.7 mm wide, 0.5 mm thick and 1 mm long (Fig. 7A, D, E). The apex of the leaves is acute and upturned, and they have a large central resin canal (170 µm in diameter; Fig. 7B, E). The vein is adaxially adjacent to the resin canal and has a flattened, abaxially concave shape in cross-sections (Fig. 7D). The vein is flanked by areas of transfusion tissue (Fig. 7B–E).

### Phylogeny

#### Living genera plus *Athrosequoia (Table 1; Fig. 8A–C)*

**Fig. 8.**
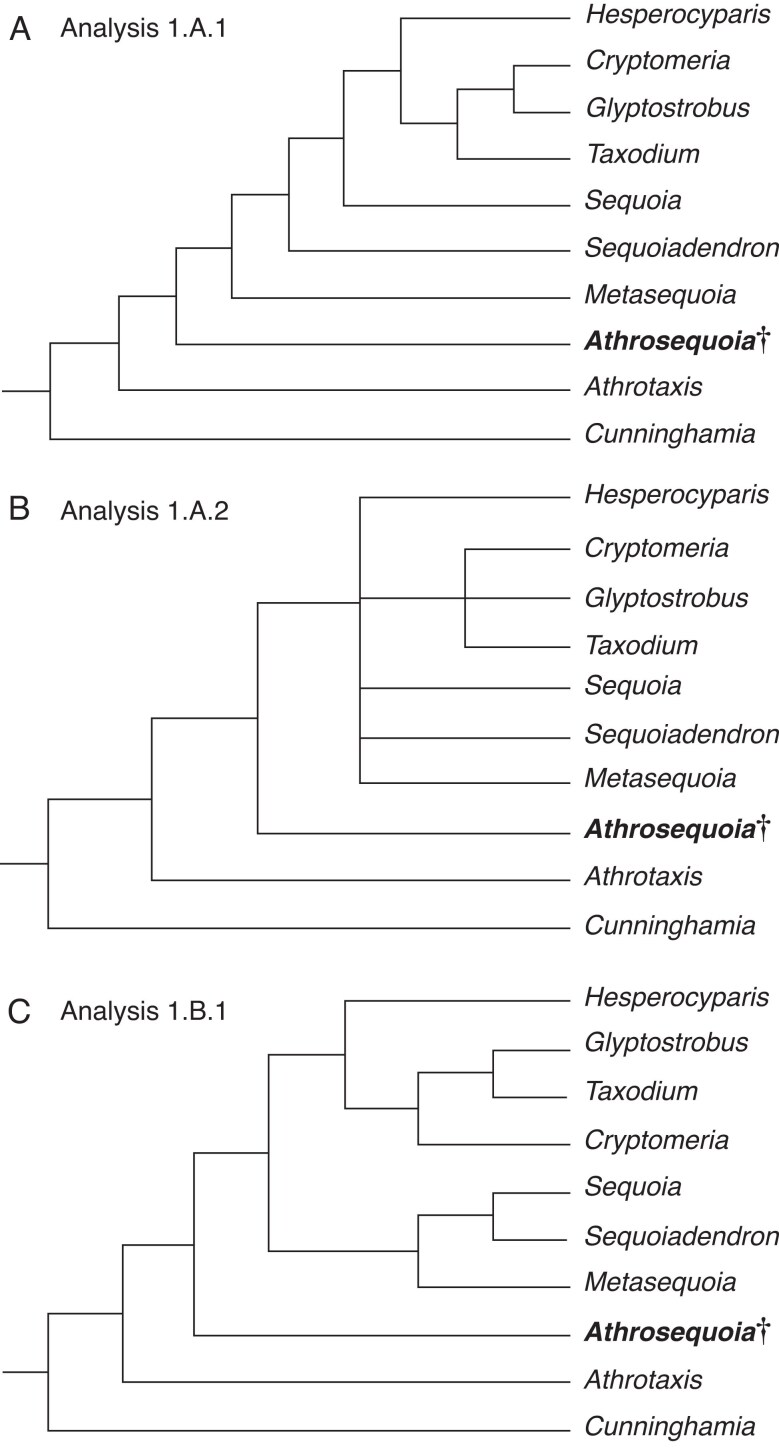
Phylogenetic relationships of living taxa and *Athrosequoia walkeri* gen. et sp. nov. (A) Single most parsimonious tree for analysis including discrete and continuous characters; Analysis 1.A.1. (B) Strict consensus tree of three most parsimonious trees for analysis that includes only discrete characters; Analysis 1.A.2. (C) Single most parsimonious tree for constrained analysis that includes discrete and continuous characters; Analysis 1.B.1. *Athrosequoia* is indicated in bold, and all fossils are marked with daggers (†).

Morphological seed cone characters consistently recover *Athrosequoia* as sister to a clade that includes the living sequoioids, taxodioids and *Hesperocyparis* (Fig. 8A, B; Analyses 1.A.1, 1.A.2 and 1.B.1). Two character state changes define the base of this clade: the central position of the pith at the base of the ovuliferous complex trace (character 32); and resin canals that do not run the entire length of the ovuliferous complex (character 47). Additionally, one continuous character (character 10; maximum number of resin canals distally in the ovuliferous complex normalized to cross-sectional area of the complex) supports this node.

*Athrosequoia* and its sister clade form a clade that is consistently recovered as sister to *Athrotaxis* (Fig. 8). Two character state changes define this node: the angle of divergence of ovuliferous complexes located midway along the cone axis is ∼90° (character 22); and the ovuliferous complex is peltate (character 23). One continuous character also supports this node (character 15; the number of resin canals at the base of the ovuliferous complex).

Constraining tree searches based on results of molecular phylogenies (Leslie *et al.*, 2012) results in trees longer that those of the unconstrained tree search by five or six steps (Fig. 8C; Analysis 1.B.1; Table 1). In the constrained topologies (Analysis 1.B.1), the sequoioids form a clade that is sister to the taxodioids + *Hesperocyparis* (Fig. 8C), in contrast to their paraphyletic configuration recovered in unconstrained analyses (Fig. 8A). Constrained topology has no effect on the position of *Athrosequoia*.

The taxodioid clade is consistently recovered as monophyletic in both the constrained and unconstrained analyses (Fig. 8) and is supported by three discrete character state changes: the angle of divergence of ovuliferous complexes located midway along the cone axis is <90° (character 22); there is a sharp angular transition between the apical region of the ovuliferous complex and the subtending region, as seen in longitudinal view (character 23); and ovuliferous complexes have lobed tips (character 28). Additionally, two continuous characters also support this node (character 2: the number of seeds per OC; and character 6: length:width aspect ratio of OC in adaxial view).

Exclusion of continuous characters collapses the sister clade of *Athrosequoia* into a polytomy, in unconstrained analyses (Analysis 1.A.2; Fig. 8B). However, continuous characters have no effect on the position of *Athrosequoia*, which does not change when they are excluded.

#### Living genera and fossils, including Athrosequoia (Table 2; Fig. 9A–C)

**Fig. 9.**
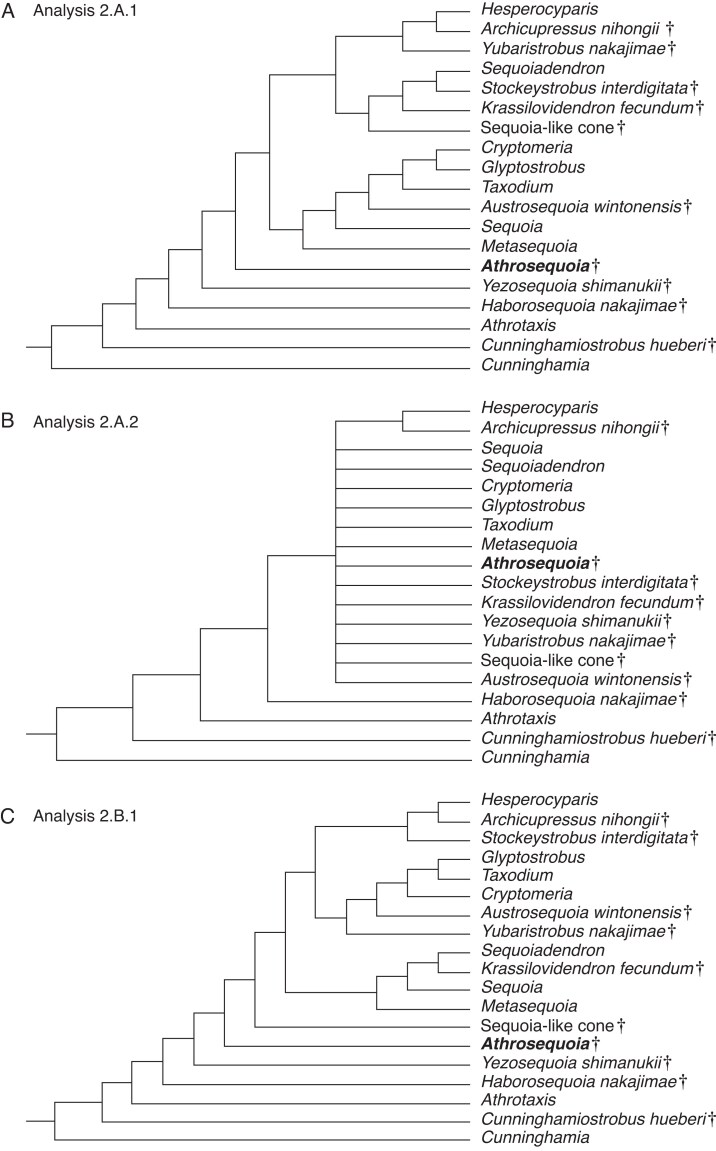
Phylogenetic relationships of living taxa, *Athrosequoia walkeri* gen. et sp. nov. and nine other fossils. (A) Single most parsimonious tree for analysis including discrete and continuous characters; Analysis 2.A.1. (B) Strict consensus tree of 40 most parsimonious trees for analysis that includes only discrete characters; Analysis 2.A.2. (C) Single most parsimonious tree for constrained analysis that includes discrete and continuous characters; Analysis 2.B.1. *Athrosequoia* is indicated in bold, and all fossils are marked with daggers (†).

The analysis based on discrete characters only resolves *Cunninghamiostrobus*, *Athrotaxis* and *Haborosequoia* as a grade basal to a large polytomy that includes all other taxa and wherein the only resolved relationship is *Archicupressus* sister to *Hesperocyparis* (Fig. 9B; Analysis 2.A.2).

**Table 2.** Summary of phylogenetic analyses of living taxa, fossil taxa and *Athrosequoia*.

	2.A.1	2.A.2	2.B.1
Character sampling	Discrete + continuous	Discrete only	Discrete + continuous
Constrained analysis	—	—	+
Tree length (number of MPTs)	118.4 (1 MPT)	94 (40 MPTs)	124.14 (1 MPT)
Living sequoioids	Polyphyletic	Part of a polytomy	Clade with *Krassilovidendron*
Living taxodioids	Monophyletic	Part of a polytomy	Monophyletic
Position of *Hesperocyparis*	Part of larger clade with sequoioid affinities	Sister to *Archicupressus* within large polytomy	Part of larger clade including taxodioids

Abbreviation: MPT = most parsimonious tree.

When included in analyses with all living genera and fossils, *Athrosequoia* is recovered above the *Athrotaxis*–cunninghamioid (*Cunninghamia* + *Cunninghamiostrobus*) grade (Fig. 9). *Haborosequoia* and *Yezosequoia* are also part of this grade that is basal to *Athrosequoia*, except for the analysis based exclusively on discrete characters (Fig. 9B; Analysis 2.A.2).

Constraining analyses that include all living genera and fossils based on the results of molecular phylogenies (Fig. 9C; Analysis 2.B.1) significantly affects the topology of the clade sister to *Athrosequoia*. In the unconstrained analysis, the sequoioids are dispersed in two clades that form a grade basal to a *Yubaristrobus–Archicupressus–Hesperocyparis* clade. In one of these two clades, *Metasequoia*, *Sequoia* and *Austrosequoia* form a grade basal to the monophyletic taxodioids; the other clade consists of *Sequoiadendron*, *Stockeystrobus*, *Krassilovidendron* and the *Sequoia*-like cone of Ohsawa *et al.* (1992*a*). Taxa with stable positions between the unconstrained and constrained analyses include: *Haborosequoia* and *Yezosequoia* as a grade above the *Athrotaxis* node; *Archicupressus* sister to *Hesperocyparis*; and *Austrosequoia* sister to the taxodioids. *Krassilovidendron* maintains a close relationship to *Sequoiadendron*. However, the positions of *Yubaristrobus*, *Stockeystrobus* and the *Sequoia*-like cone of Ohsawa *et al.* (1992*a*) change drastically between the constrained and unconstrained analyses (Fig. 9A, C; Analyses 2.A.1. and 2.B.1).

## DISCUSSION

### *Athrosequoia* is an early member of taxodiaceous Cupressaceae

*Athrosequoia walkeri* is represented by a single anatomically preserved cone from the Budden Canyon Formation of California. The morphology of ovuliferous complexes places this cone among the Cupressaceae, a group also represented in the same fossil assemblage by foliage and pollen cones (Ortiz and Tomescu, 2014). *Athrosequoia* is the oldest member of the Cupressaseae that possesses peltate ovuliferous complexes. This genus combines morphological and anatomical characters of living athrotaxidoids, sequoioids and taxodioids. Thus, *Athrosequoia* shares with *Athrotaxis* the adaxially eccentric pith of the ovuliferous complex trace (Fig. 5A–C), the adaxially shifted trajectory of the vasculature as seen in longitudinal sections of ovuliferous complexes (Fig. 5D, E) and the absence of a resin canal at the base of the ovuliferous complex. Furthermore, *Athrosequoia walkeri* is similar to *Athrotaxis cupressoides*, which has peltate ovuliferous complexes with ∼90° divergence angle (see below for further discussion on the species of *Athrotaxis* used in the phylogenetic study). Similarities with the sequoioids include the peltate shape of ovuliferous complexes, the 90° divergence angle of the complexes and a horizontal groove on the peltate head of ovuliferous complexes (Fig. 5F). The horizontal groove in *Athrosequoia* is shallower and contains a thicker mucro than those observed in sequoioids. A similar mucro shape is found in *Athrotaxis cupressoides*, in which the mucro is, however, not sunken into a horizontal groove. Like *Sequoia*, *Athrosequoia* has a parenchymatous layer surrounding the sclerenchymatous tissues of ovuliferous complexes in mature undehisced cones (see below; Fig. 6C). *Athrosequoia* has taxodioid crossfield pitting and, similar to the taxodioids, the vasculature is significantly shifted adaxially within the ovuliferous complex (Fig. 5D, E). In summary, *Athrosequoia* is unique in combining peltate ovuliferous complexes with a horizontal groove (a sequoioid trait), with features of other taxodiaceous Cupressaceae (such as adaxial eccentric pith of ovuliferous complex traces and a more robust mucro, as seen in *Athrotaxis*). This novel combination of characters excludes *Athrosequoia* from all known genera of the Cupressaceae, supporting the erection of a new genus, and implies that this new genus fits among the taxodiaceous Cupressaceae.

Fossil taxa, such as *Athrosequoia*, which combine features of several living taxa, are key to understanding the evolution of the groups to which they belong, by demonstrating putative intermediate morphologies between previously identified genera (Mongiardino Koch and Parry, 2020). However, to date, the incomplete sampling of morphological seed cone characters has precluded achieving resolution at a finer taxonomic scale, despite broad consensus that seed cones are the most informative structures in phylogenetic analyses of conifers (Rothwell *et al.*, 2011; Escapa and Catalano, 2013; Smith *et al.*, 2016; Herrera *et al.*, 2017). This is especially important for Cupressaceae, whose seed cone morphology and anatomy are variable in characters that do not vary in other conifer families (e.g. number of seeds). Therefore, we urgently need to explore the anatomy and morphology of seed cones with the goal of constructing characters that can describe their intricacies and variation present in different taxa. *Athrosequoia* and the novel combination of characters it represents, especially in light of its age, provide some insights about evolution at the base of the Cupressaceae.

### Taphonomy and ontogenetic stage of the cone

The anatomical preservation of the *Athrosequoia* cone allows for comparisons with living Cupressaceae cones that inform the ontogenetic stage of this fossil cone. The parenchymatous layer consisting of radially flattened cells that surrounds most parts of the cone (Fig. 6D) is comparable to similar layers observed around *Sequoia* cones (Fig. 6C). Because of the incomplete preservation of this layer, which appears only as a halo around most cone parts, we initially considered the alternative hypothesis that it might represent a taphonomic (diagenetic) artefact, such as a layer of different mineralogy precipitated adjacent to the plant tissues as a result of their chemistry in interaction with permineralizing solutions. However, we reject this alternative explanation owing to: (1) the absence of this layer from the leaves attached to the cone peduncle, which underwent the same taphonomic processes; (2) the conspicuous cellular nature of this layer, where it is well preserved; and (3) its close similarity to living counterparts.

In *Sequoia*, the external parenchymatous layer is present only in nearly mature but still green cones. This indicates that the *Athrosequoia* cone was still green and had not reached full maturity. However, the high degree of sclerification of the ovuliferous complex tissues demonstrates that the cone had reached mature size. The absence of seeds suggests that the cone had dehisced prior to fossilization, whereas the presence of green tissue around it (associated with immature stages) is inconsistent with this interpretation and points to taphonomic loss of the seeds. It is also possible that the cone, while still retaining partly a green layer, had already started dehiscing, and that taphonomic factors quickened the detachment and dispersal of the seeds.

Another notable difference between *Athrosequoia* and the taxodiaceaous Cupressaceae is the small size of the ovulate cone of the former, which, nevertheless, has more than ten ovuliferous complexes. Seed cones of similar or smaller size are seen in several genera of the Cupressoideae and Callitroideae subfamilies (e.g. *Juniperus*, *Diselma*, *Microbiota* and *Chamaecyparis*), but all have much fewer ovuliferous complexes, with opposite or whorled taxis. It is also worth noting that at the pollination stage the seed cones of Cupressaceae can have roughly similar sizes to those of *Athrosequoia* [see data from Huntsman and Leslie (2024) for size variation of pollination stage cones in Cupressaceae]. However, pollination-stage cones throughout the family have distinctive leaf-like ovuliferous complexes (Huntsman and Leslie, 2024), unlike *Athrosequoia*, which has peltate ovuliferous complexes and whose anatomy denotes maturity. Additionally, when considering the fossil record, *Athrosequoia* is not unique, because several similarly sized fossil seed cones are known. For example, *Austrosequoia wintonensis* ranges from 9 to 16 mm in length and contains 29–49 ovuliferous complexes (Peters and Christophel, 1978). Given the higher diversity of taxodiaceous Cupressaceae in the fossil record in comparison to modern diversity, it is not surprising that there is higher morphological disparity among them than documented in living representatives, including in terms of cone size.

### Exploring the role of fossils and seed cone morphology in Cupressaceae phylogeny

The main objective of our phylogenetic experiments was to explore new sources of data for morphological character construction in order to gain insights into the phylogenetic placement of *Athrosequoia*, within the broader context of relationships between living and extinct Cupressaceae, as resolved by previous studies. Specifically, our analyses experimented with taxon and character inclusion/exclusion, in addition to topological constraints. Here, we discuss: (1) the position of *Athrosequoia* in relationship to extant taxa and the impact of our new morphological seed cone characters on these relationships; (2) the effect of fossils on the relationships of extant cupressaceous conifers; and (3) the implications of relationships between extant and fossil taxa for the systematic placement of the latter. In all instances, we explore the influence of seed cone morphological characters by comparing the topologies obtained in analyses constrained based on molecular data with those obtained in unconstrained analyses. Overall, our experiments reveal the importance of characters that capture as much comparative morphological and anatomical variability of seed cones as possible for resolving relationships within clades that have a deep fossil history.

#### Position of *Athrosequoia* and influence of the new seed cone characters on phylogenetic hypotheses

Seed cone characters support a stable placement of *Athrosequoia* with respect to the living Cupressaceae genera, irrespective of character sampling (discrete only or discrete + continuous), inclusion/exclusion of fossils and topological constraints based on molecular phylogenies (Figs 8 and 9; Tables 1 and 2). All the analyses place the divergence of *Athrosequoia* between that of *Athrotaxis* and the divergence of living sequoioids. This position could be construed to indicate that the seed cone morphology of *Athrosequoia* is intermediate between the morphology of *Athrotaxis* and that of members of the clade that includes the rest of the family. When added to the full taxon sampling (extant + fossil), *Athrosequoia* maintains the same stable position with respect to the extant genera as in the analyses that exclude all other fossils (Analysis 1).

Our morphological characters recover relationships that are congruent with those of molecular analyses for taxodioids, but not for the sequoioids. This is immediately apparent in analyses that include only living genera plus *Athrosequoia*. In those analyses, the taxodioids are monophyletic irrespective of topological constraints. However, when seed cone characters alone are used (i.e. unconstrained analyses; Analyses 1.A.1 and 1.A.2), the sequoioids are resolved as paraphyletic at the base of taxodioids + *Hesperocyparis* (Fig. 8A; Table 1) or in a polytomy with the taxodioid clade and *Hesperocyparis* (Fig. 8B). This inconsistency notwithstanding, it is important to note that our morphological characters converge on the same general pattern of relationships among major cupressaceous subfamilies as recovered in molecular phylogenies. In this context, the contrast in resolution between the unconstrained trees obtained without and with continuous characters indicates that the latter parallel the resolving power of molecular data, for this set of taxa. This is consistent with the idea that the morphology of living taxa mirrors the pattern of diversification of subfamilies, i.e. subfamilies are clearly distinct morphologically (Leslie *et al.*, 2018).

#### Morphological variety in *Athrotaxis*

The morphological similarities between *Athrosequoia* and *Athrotaxis* bring into light the morphological diversity of seed cones in the genus *Athrotaxis*. Notably, the ovuliferous complexes of *Athrotaxis cupressoides* are distinctively peltate, whereas those of *Athrotaxis selaginoides* are inflated distally but are not peltate. These two species also differ in the divergence angle of mature ovuliferous complexes from the cone axis. *Athrotaxis laxifolia* is a hybrid of *Athrotaxis cupressoides* and *Athrotaxis selaginoides* (Isoda *et al.*, 2000; Worth *et al.*, 2016), and the morphologies of *Athrotaxis laxifolia* vary and are often intermediate between *Athrotaxis cupressoides* and *Athrotaxis selaginoides*. Therefore, our morphological matrix does not capture the polymorphism present within the genus. The seed cones of *Athrotaxis laxifolia* used for this study are more similar to *Athrotaxis selaginoides* than to *Athrotaxis cupressoides*. For the distal end of the ovuliferous complex (character 28), in the material that we examined the inflated terminal adaxial region that develops to touch the ovuliferous complexes above it is slightly lobed (see also Jagel and Dörken, 2014: fig. 2B of *Athrotaxis laxifolia*). This scoring depends on the *Athrotaxis* species included and the way one perceives the lobing of structures, hence the character should be re-coded in analyses that include several species of *Athrotaxis*. Nevertheless, in our analysis this character does not appear to have implications for the placement of *Athrotaxis*, because it is not forcing its inclusion among the taxodioids.

#### Phylogenetic relationships and taxonomic affinities of fossils in light of the new seed cone characters

When fossils are included in analyses, they are distributed amongst the living genera (Fig. 9). The positions of the fossils provide clues to their possible taxonomic affinities. For some of the fossils, these clues are consistent with hypotheses of affinities discussed in previous studies, whereas for others they are at odds with previously published hypotheses. For instance, *Haborosequoia* and *Yezosequoia* diverge at intermediate nodes between *Athrotaxis* and *Athrosequoia*, irrespective of whether relationships are constrained based on results of molecular phylogenies, which suggests that these two genera might not be *bona fide* sequoioids (Fig. 9).

*Krassilovidendron* maintains a close relationship to *Sequoiadendron*, consistent with its suggested taxonomic placement among the sequoioids (Sokolova *et al.*, 2017). In the unconstrained analysis including fossils + extant taxa and all characters (Fig. 9; Analysis 2.A.1), this relationship is supported by the geometry of the abaxial component of the distal ovuliferous complex, which forms a straight line (character 42); two continuous characters also exhibit notable changes at this node (character 3: number of ovuliferous complex per cone; and character 14: the surface area of xylem at the base of the ovuliferous complex normalized to the cross-sectional area of the complex at the base).

Other stable relationships with respect to constraints based on molecular phylogenies are *Archicupressus* sister to *Hesperocyparis* and *Austrosequoia* sister to the taxodioids (Fig. 9A, C; Analyses 2.A.1 and 2.B.1). These results confirm the proposed cupressoid affinities of *Archicupressus* (Ohsawa *et al.*, 1992*b*). The stable position of *Austrosequoia* sister to the living taxodioids (supported by <90° angle of divergence of ovuliferous complexes) suggests taxodioid affinities for *Austrosequoia*, which was previously compared with *Athrotaxis* and the sequoioids (Peters and Christophel, 1978) and was considered a probable stem group of the monophyletic Sequoioideae (Mays *et al.*, 2017). In this context, it is worth noting that the scoring of *Austrosequoia* is incomplete (only 13 discrete characters scored) and should be reinvestigated. The fossil material is well preserved and could be scored more precisely for many of the characters, such as angle of divergence of ovuliferous complexes, seed characters and many of the anatomical characters. With reinvestigation, this could shift the placement of *Austrosequoia* to other subfamilies (such as sequoioids), with greater support. For *Yubaristrobus*, the taxodioid affinities suggested by Ohsawa *et al.* (1993) are supported in our constrained analyses (Fig. 9C; Analysis 2.B.1). However, this is in contrast to its position obtained in the unconstrained analyses (Fig. 9A; Analysis 2.A.1).

#### Importance of improved morphological character sampling in seed cones

Our set of morphological characters recovers broadly the same relationships among living genera (Fig. 8A; Analysis 1.A.1) as resolved by all molecular analyses (Gadek *et al.*, 2000; Kusumi *et al.*, 2000; Quinn *et al.*, 2002; Leslie *et al.*, 2012; Mao *et al.*, 2012; Yang *et al.*, 2012 trees based on *matK* and *rps3*) and by the morphology-based analysis of Schulz and Stützel (2007). The sequoioids are the exception; they are resolved as a clade in molecular analyses but as a paraphyletic group by our characters. Nevertheless, they are still positioned crownward of *Athrotaxis* and rootward of the taxodioids + cupressoids clade, similar to their placement in molecular analyses.

Of the previously published studies that include fossils, many are focused on taxa of the cunninghamioid plexus that are positioned rootward of the bulk of taxa included in our analyses (Escapa *et al.*, 2008; Rothwell *et al.*, 2011; Shi *et al.*, 2014; Herrera *et al.*, 2017; Dong *et al.*, 2018; Atkinson *et al.*, 2021). These studies use variations on Farjon’s (2005) characters and recover polytomies that do not resolve the relationships between *Athrotaxis*, sequoioids, taxodioids and cupressoids. An analysis by Ohsawa (1997) includes 25 taxa, of which 8 are fossil, and ten seed cone characters. Six of Ohsawa’s fossil species are also included in our analyses. Of these, Ohsawa recovered *Archicupressus* closely related to the cupressoids and *Yubaristrobus* sister to the *Archicupressus* + cupressoid clade, as in our Analysis 2 (Fig. 9A; Analysis 2.A.1). Also similar to Ohsawa’s results, *Haborosequoia*, whose position is not fully resolved in Ohsawa’s analysis, diverges crownward of *Athrotaxis* in our results. Two fossils, *Yezosequoia* and the *Sequoia*-like cone of Ohsawa *et al.* (1992*a*), occupy positions that are incongruent between Ohsawa’s (1997) and our analyses. Nevertheless, the *Sequoia*-like cone is associated with other sequoioids in both analyses. The position of *Cunninghamiostrobus*, resolved as diverging rootward of *Cunninghamia* by Ohsawa (1997), is constrained in our analysis by the use of *Cunninghamia* as the outgroup.

The above comparisons show that improved sampling of morphological characters in seed cones (i.e. 60 characters, as opposed to a maximum of 25 used in previous studies; Farjon, 2005) can resolve relationships among cupressaceous subfamilies broadly congruent with the results of molecular data. Thus, densely sampled seed cone characters show promise in generating robust hypotheses of phylogenetic placement of fossil species. These hypotheses are augmented by including constraints on relationships based on the results of molecular phylogenies.

The new fossil conifer, *Athrosequoia walkeri*, adds to the diversity of cupressaceous plants known from the Cretaceous. This fossil provides insight into the morphological evolution of the family and the diversity of the family during the Early Cretaceous. Notably, *Athrosequoia* is the oldest cupressaceous cone with peltate ovuliferous complexes and possesses a combination of features characteristic of several taxodiaceous Cupressaceae subfamilies: living athrotaxidoids, sequoioids and taxodioids. Documenting and making sense of such mosaicism that is common among Mesozoic conifers, within a phylogenetic framework, provides the only path towards elucidating patterns of morphological evolution in the family (Atkinson *et al.*, 2021).

Our study also uses the most extensive sampling of discrete and continuous morphological seed cone characters in the Cupressaceae, to date. The results of our exploratory phylogenetic analyses based on these characters and on an extensive sampling of fossil taxodiaceous Cupressaceae highlight the power and also the limitations that seed cone characters, considered in isolation, bring to elucidating the phylogenetic position of fossil species known exclusively based on this organ, in addition to elucidating phylogenetic relationships, in general. For taphonomic and biological reasons, seed cones are among the most common organ found in the fossil record for most conifer families. Therefore, alongside discovery and in-depth description of new fossils, future efforts should expand morphological character construction by detailed description of the morphology and anatomy of seed cones in extant taxa. Such in-depth exploration of morphological characters can provide tools both for palaeobotany and for systematics in helping to address phylogenetic relationships and providing reliable time constraints for dating molecular analyses. Future steps along these paths should include the expansion of our morphological matrix in taxa, in addition to characters that sample the morphology of all plant organs. Such detailed descriptive data, combined with tree topology constraints obtained from molecular phylogenetics, provide the best opportunities to understand the phylogenetic position of extinct cupressaceous seed cones, and therefore, morphological evolution in the family.

## Supplementary Material

mcaf099_Supplementary_Data

## SUPPLEMENTARY DATA

Note: taxa and characters included in phylogenetic analyses, with sources of information for character scoring. Supplementary Data Sheet S1: discrete character scorings (nexus file). Supplementary Data Sheet S2: raw values (before bracketing and normalization) of continuous characters (Excel file). Supplementary Data Sheet S3: final phylogenetic matrix with discrete and continuous character scorings (TNT file). Supplementary Data Sheet S4: zipped folder with tree files for the six analyses (1.A.1, 1.A.2, 1.B.1, 2.A.1, 2.A.2 and 2.B.1) (tree files).
