# Supplementary material for: Expanded character sampling inspired by a new Cretaceous conifer seed cone from California: importance of morphology in resolving relationships among the Cupressaceae
Source: Ann Bot. 2025 Jun 4;137(6):1902–18. doi: 10.1093/aob/mcaf099 (PMC13274983; doi:10.1093/aob/mcaf099)
Supplement: mcaf099_Supplementary_Data [file mcaf099_supplementary_data.zip › PfeilerEtAl2024_SupplNote.docx]

**Expanded character sampling inspired by a new Cretaceous conifer seed cone from California: importance of morphology in resolving relationships among the Cupressaceae**

Kelly Pfeiler^1,2,^*, Alexander C. Bippus^3^, Ashley Ortiz^3^, Ashley R. Kammet ^3^, Ignacio H. Escapa^4^, Alexandru M.F. Tomescu^3,^*

^1^ Department of Ecology and Evolutionary Biology, University of Kansas, Lawrence, Kansas 66045, USA

^2^ Biodiversity Institute, University of Kansas, Lawrence, Kansas 66045, USA

^3^ Department of Biological Sciences, California State Polytechnic University Humboldt, Arcata, California 95521, USA

^4^ Consejo Nacional de Investigaciones Científicas y Técnicas and Museo Paleontológico Egidio Feruglio, Trelew 9100, Chubut, Argentina

**Supplementary Note.** Taxa and characters included in phylogenetic analyses; with sources of information for character scoring.

**Taxon sampling**

Extant taxa

*Athrotaxis laxifolia* (Farjon 2005; our observations)

*Cryptomeria japonica* (Farjon 2005; our observations)

*Cunninghamia lanceolata* (Florin 1951; Farjon 2005; Schulz & Stützel 2007; our observations)

*Hesperocyparis (Cupressus) macrocarpa* (Farjon 2005; our observations)

*Glyptostrobus* (Farjon 2005; our observations)

*Metasequoia* (Farjon 2005; our observations)

*Sequoia sempervirens* (Farjon 2005; our observations)

*Sequoiadendron giganteum* (Farjon 2005; our observations)

*Taxodium distichum* (Farjon 2005; our observations)

Fossil taxa

*Archicupressus nihongii* (Ohsawa et al. 1992b)

*Athrosequoia walkeri* (our observations)

*Austrosequoia wintonensis* (Peters & Christophel 1978)

*Cunninghamiostrobus hueberi* (Miller 1975)

*Haborosequoia nakajimae* (Ohsawa et al. 1992b)

*Krassilovidendron fecundum* (Sokolova et al. 2017)

*Sequoia*-like cone (Ohsawa et al. 1992a)

*Stockeystrobus interdigitata* (Rothwell & Ohana 2016)

*Yezosequoia shimanukii* (Nishida 1991)

*Yubaristrobus nakajimae* (Ohsawa et al. 1993)

**Character construction**

Continuous characters

0. Cone size (length = L)


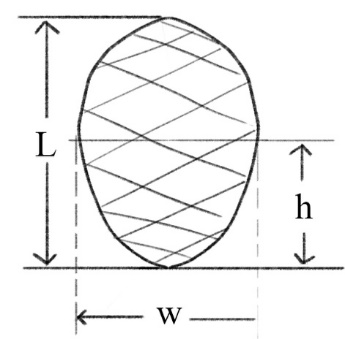


1. Cone Length:Width ratio = length:max. width ratio (L:W)


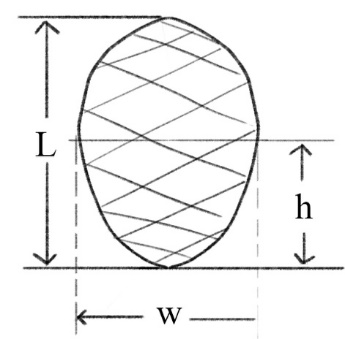


2. Number of seeds per ovuliferous complex

3. Number of ovuliferous complexes per cone

4. Position of cone’s widest diameter, from base (h:L)


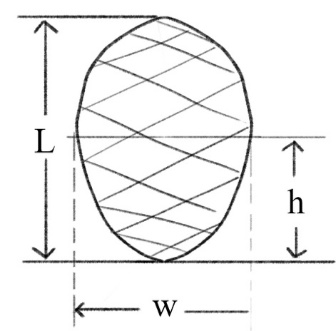


5. Basal cone axis diameter:narrowest size of ovuliferous complex stalk/base (x:y). Measure overall diameter of cone axis at the base of the cone (i.e. immediately below the basal-most ovuliferous complexes) and divide by the size of the ovuliferous complex at the base. The minimum size of the ovuliferous complex at the base was measured in longitudinal cone sections for most cones, but in a few (fossil) cases where only cone cross sections were available, it was measured in those sections.


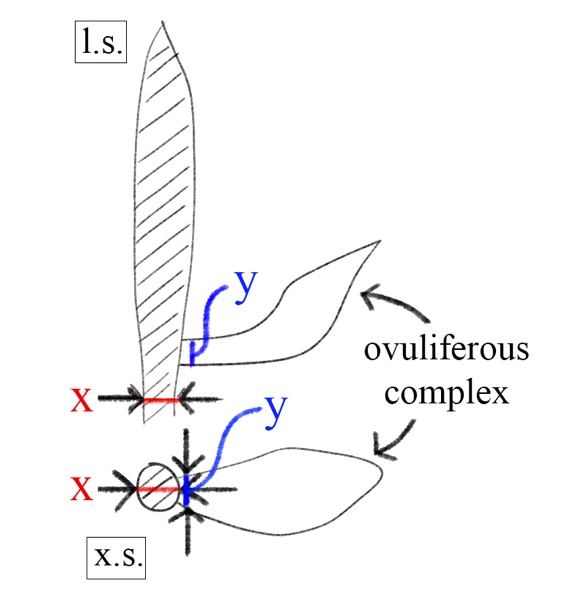


6.Ovuliferous complex aspect ratio in adaxial view = length:max. width ratio (L:W)


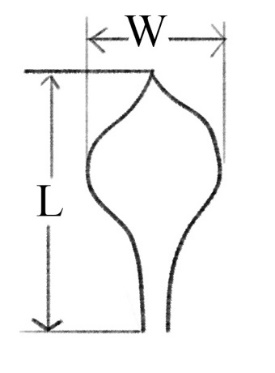


7. Position of ovuliferous complex’s widest point, from the base of the ovuliferous complex (l:L)


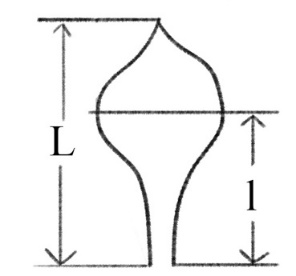


8. Maximum number of bundles in the ovuliferous complex (distally) normalized to cross-sectional area of ovuliferous complex (at its widest). Count highest number of distinct vascular bundles that can be observed in any cross section of an ovuliferous complex and divide by the cross-sectional surface area of the ovuliferous complex measured at its widest point; i.e., number of vascular bundles per mm^2^.

9. Xylem diameter at base of ovuliferous complex:overall cone axis length. The xylem diameter at base of the ovuliferous complex was measured in any plane of section available (longitudinal cone sections or basal-most serial cross-sections of ovuliferous complexes).


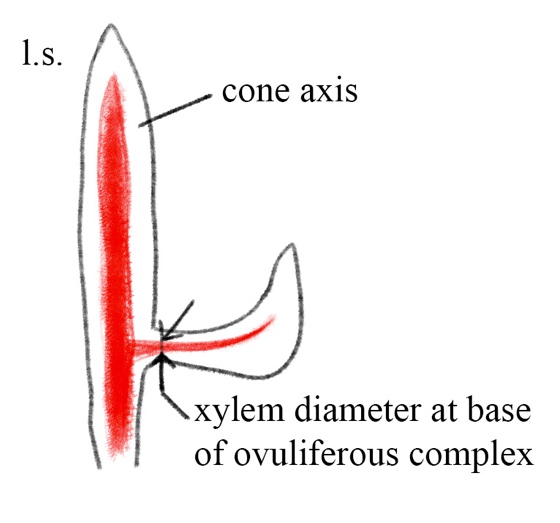


10. Maximum number of resin canals in the ovuliferous complex (distally) normalized to cross-sectional area of ovuliferous complex (at its widest). Count highest number of distinct resin canals that can be observed in any cross section of an ovuliferous complex and divide by the cross-sectional surface area of the ovuliferous complex measured at its widest point; i.e., number of resin canals per mm^2^.

11. Cone axis slenderness = max. cone width: max. thickness of cone axis

12. Cone axis xylem slenderness = cone axis radius:axis xylem radius (measured at widest point of cone)

13.Ovuliferous complex basal width aspect = max. width of ovuliferous complex:width of ovuliferous complex at base (W:µ). These were measured in adaxial views of the ovuliferous complexes or in cone cross sections (for fossils)


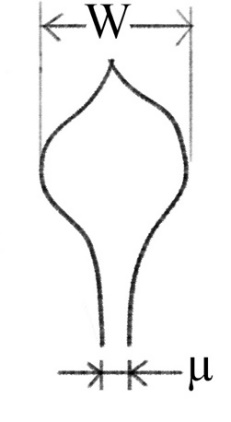


14. Basal ovuliferous complex xylem slenderness = cross-sectional surface area of xylem at base of ovuliferous complex:overall cross-sectional surface area of ovuliferous complex at base

15. Number of resin canals at the base of ovuliferous complex

16. Resin canal thickness at base of ovuliferous complex = cross-sectional surface area of resin canal at base of ovuliferous complex:overall cross-sectional surface area of ovuliferous complex at base

Discrete characters

17. Seed cone attachment

0 = terminal; 1 = lateral

18. Seed cone grouping

0 = solitary; 1 = clustered

19. More than one growth ring in cone axis

0 = absent; 1 = present

20. Vascular cylinder in cone axis

0 = complete; 1 = dissected

21. Taxis of ovuliferous complexes

0 = alternate/helical; 1 = opposite/whorled

22. Angle of ovuliferous complex divergence from cone axis

0 = mostly 90; 1 = mostly <90

This character was scored for mature cones that were not dehisced or immediately after dehiscence (i.e. before cones like *Taxodium* disarticulate at full maturity). There is variation along the cone axis in the angle of divergence of ovuliferous complexes and that is why the character was scored based on ovuliferous complexes in the median region of the cones.

23. Sharp transition in angle between distal region of ovuliferous complex (exposed at cone surface) and subtending region of ovuliferous complex as seen in side view

0 = absent; 1 = present; 2 = peltate

Rather than strictly differentiating between peltate and non-peltate ovuliferous complexes, this character splits the gradient between foliate and peltate morphologies into three states. Character 23, along with character 27 is aimed at capturing what most matrices separate into “foliate,” “peltate,” or “sub-peltate/cuneate/valvate” ovuliferous complex shape.


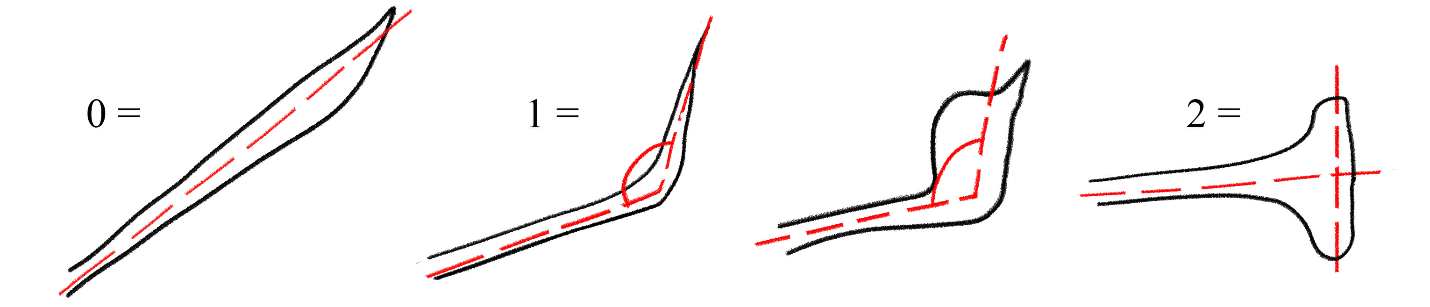


24. Horizontal groove on peltate head

0 = absent; 1 = present

This character is coded on mature cones, and we advise it not be scored for cones where maturity stage is early or ambiguous (like in some fossils).

25. Mucro/”Bract” tip size of ovuliferous complexes in mature cone

0 = absent; 1 = small, acute (e.g. *Sequoia*); 2 = elongated (e.g. *Cryptomeria*); 3 = not pronounced (e.g. *Taxodium*)

26. Mucro/”Bract” tip in mature cone; vascularization

0 = absent; 1 = present

27. Distal part of ovuliferous complex significantly thicker than base (side view)

0 = absent; 1 = present

28. Distal end of ovuliferous complex with lobed tips

0 = absent; 1 = present

Here, distal end refers to the part of the ovuliferous complex adjacent to, and that develops to rest against, the ovuliferous complex(es) above it. “sealing” the fertilized ovules inside the cone. The character is scored for fully mature cones and applies to fully developed features of the ovuliferous complex (i.e., not the vestigial flaps of *Cunninghamia* and *Taiwania*). In the *Athrotaxis* material that we examined the inflated terminal adaxial region of the ovuliferous complex that develops to touch the ovuliferous complexes above it is slightly lobed [see also Figure 2B of *A. laxifolia* in Jagel and Dörken (2014)]. This scoring depends on the *Athrotaxis* species included and the way one perceives the lobing of structures, so the character should be re-coded in analyses that include several species of *Athrotaxis*.

29. Shape of lobed tips of the distal end of the ovuliferous complex

0 = round; 1 = protruding, pointed

30. Degree of fusion of ovuliferous complexes (i.e., fusion of bract and ovuliferous scale)

0 = no fusion; 1 = fusion in any degree

If bract and ovuliferous scale are fused to any degree, this character is scored as present. All taxa in Cupressaceae have some degree of fusion between bract and ovuliferous scale (=present) regardless of homology interpretations in the family. This character will be useful to differentiate Cupressaceae from other extinct and extant conifers if they are included in future analyses with a broader taxon sampling.

31. Symmetry of ovuliferous complex trace at origin

0 = radial (cylindrical); 1 = bilateral (dorsiventral)

32. Symmetry of radial ovuliferous complex trace

0 = pith central; 1 = pith adaxially eccentric; 2 = pith abaxially eccentric


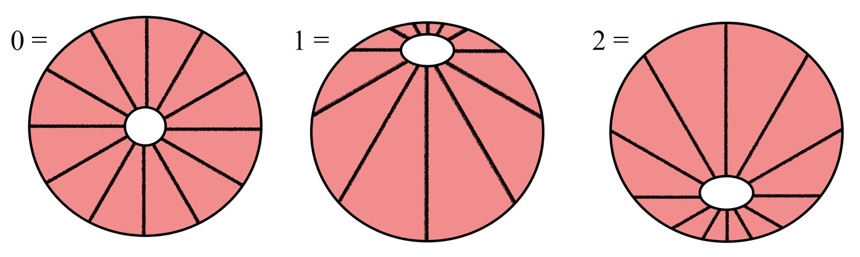


33. Basal-most dissection of ovuliferous complex trace

0 = forms bundle that diverges adaxially; 1 = forms bundle that diverges abaxially


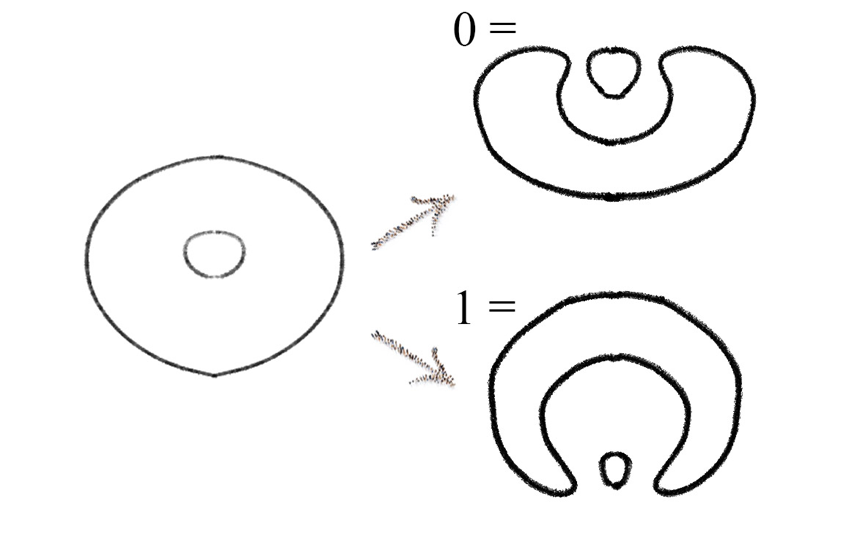


34. Ovule vascular supply

0 = supplied by first-diverging bundle of ovuliferous complex trace; 1 = supplied by secondary or higher order bundles diverging from ovuliferous complex vasculature

35. Basal-most diverging bundle (if abaxial) unbranched

0 = absent (bundle branched); 1 = present (bundle not branched)

If the first bundle to diverge is adaxial (i.e., character 33 = 0) then this character is scored as inapplicable (“-“).


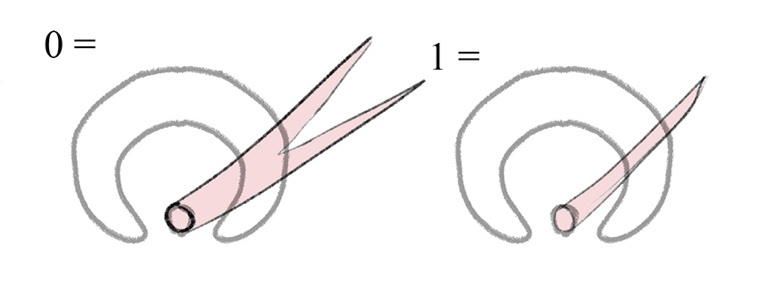


36. Ovuliferous complex trace diverges to form:

0 = line of “abaxial” bundles [*Cunninghamia*]; 1 = “ring” of bundles

This character is observed close to the base of the ovuliferous complex. An “abaxial” bundle is one that has xylem situated above the phloem. In a “ring” of bundles, the phloem is oriented toward away from the central axis of the ovuliferous complex, so that the bundles located on the adaxial side of the “ring” correspond to the “inverted bundles” of Tomlinson and Takaso (2002). This character is not related to character 33, as it refers to the vascular supply of the ovuliferous complex other than the basal-most diverging bundle (if present). In general, this character is associated with ovuliferous complexes that are foliate (= 0; line of abaxial bundles) versus those that are not (= 1; ring of bundles) in Cupressaceae.


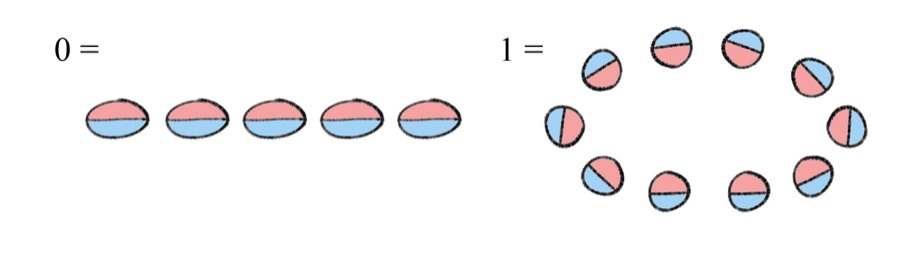


37. Distal divergences of ovuliferous complex vasculature form:

0 = adaxial and abaxial files of bundles [where adaxial file of bundles refers to the “inverted bundles” of Tomlinson and Takaso (2002)]; 1 = continuous “ring” of bundles; 2 = adaxial file of bundles + abaxial ring of bundles [*Stockeystrobus*]

In the distal region of the ovuliferous complexes the adaxial bundles and the abaxial bundles can either form distinct files, a ring, or an upper file and an abaxial ring (see diagram). Character 37 is scored as inapplicable for taxa that do not have “adaxial” bundles (i.e., bundles that have phloem above the xylem) in the vasculature of distal regions (character 36 = 0; e.g., *Cunninghamia*, with a single file of “abaxial” bundles).


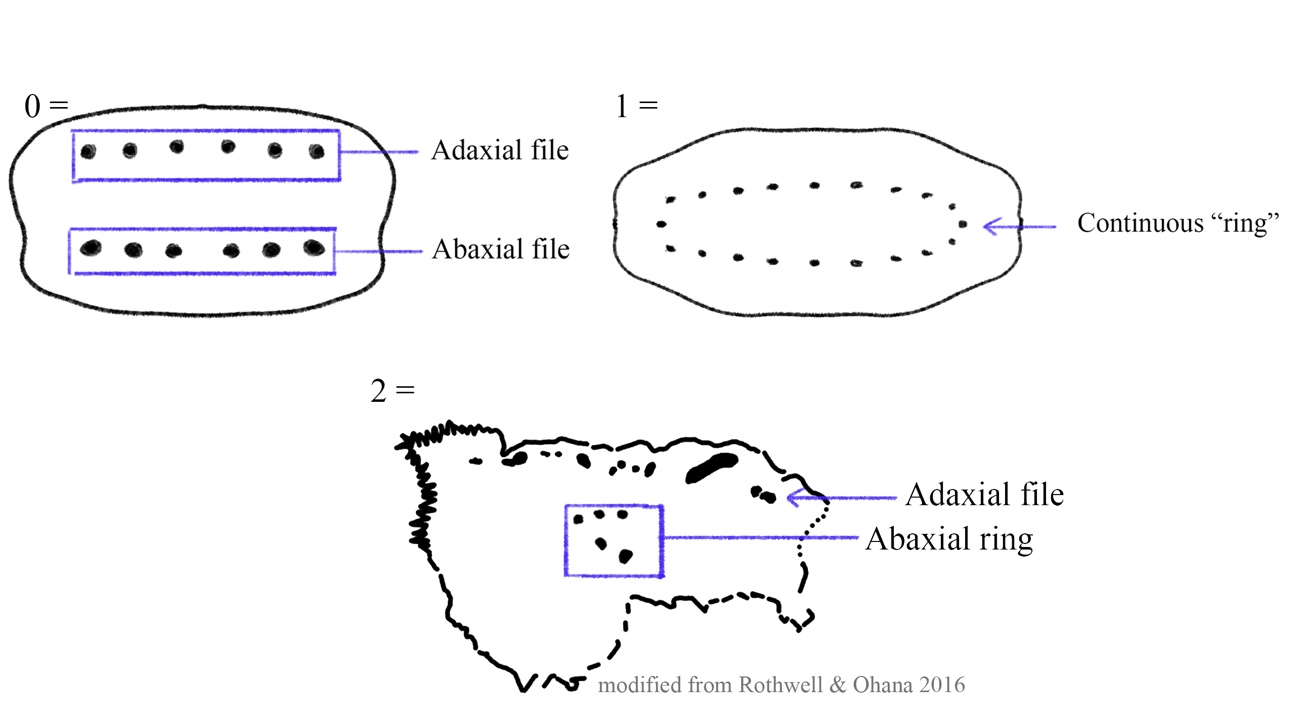


38. Vertical positioning of vasculature (as a whole) within ovuliferous complex, distally (where it is dissected)

0 = centered; 1 = slightly shifted adaxially (i.e., most or all of it above median plane, <1/3); 2 = shifted adaxially a lot (>1/3, i.e., in the upper third of the ovuliferous complex height, as seen in cross section)


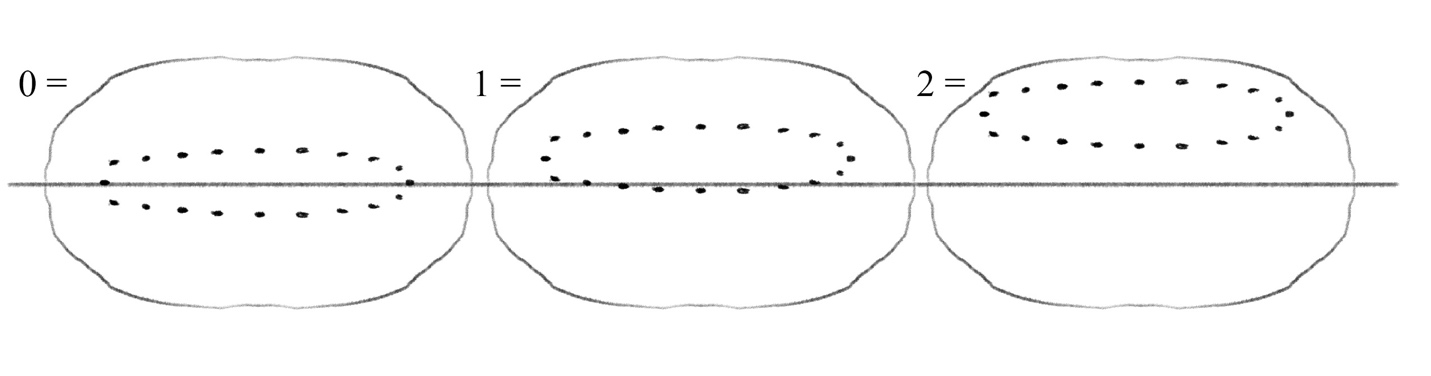


39. Adaxial component of ovuliferous complex vasculature supplies:

0 = exclusively the ovules; 1 = ovuliferous complex beyond the ovules

In taxa like *Cunninghamia* the bundles of the adaxial component, which have short trajectories, supply the ovules only (= 0; see diagram below and Figure 5E in main text). In other taxa the adaxial bundles reach beyond the ovules (see diagram below and Figure 5F in main text)


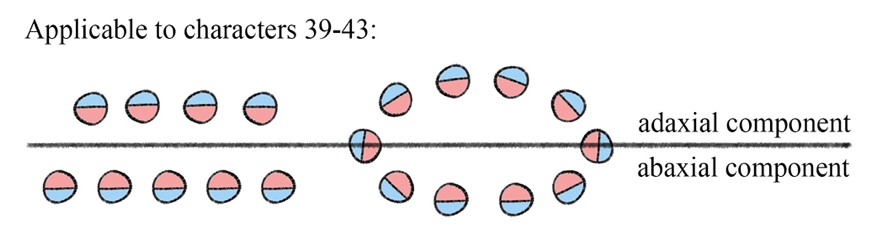


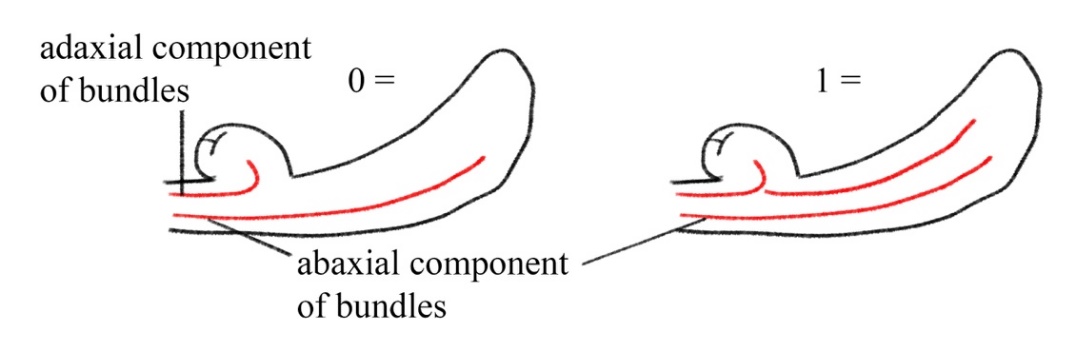


40. Adaxial component of distal ovuliferous complex vasculature forms [distal = where it is highly branched, closer to the tip than the base of the complex]:

0 = adaxially convex shape, as seen in cross section of ovuliferous complex; 1 = linear horizontal file; 2 = adaxially concave shape

The adaxial component of the vasculature, consisting mainly of bundles with phloem above the xylem, is referred to by some authors [e.g., Tomlinson and Takaso (2002)] as “inverted bundles”. This character is scored inapplicable (“-“) for taxa that do not have an adaxial component of bundles (i.e., taxa in which character 36 = 0, like *Cunninghamia*).


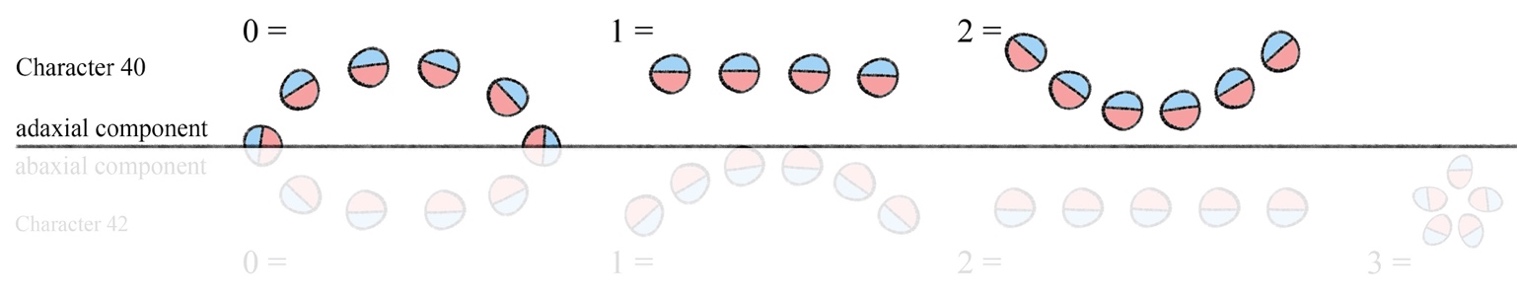


41. Abaxial component of ovuliferous complex vasculature runs:

0 = the entire length of the ovuliferous complex; 1 = only part of the ovuliferous complex length (except for mucro bundle, where present)

Abaxial component of the vasculature generally refers to the vascular bundles that have xylem above phloem. These bundles can run for the entire length of the ovuliferous complex (= 0) or can be shorter and not reaching the distal-most parts of the ovuliferous complex. This character does not refer to the vascular tissue that supplies the mucro, so if scoring this character from longitudinal, care needs to be taken to avoid scoring the sagittal section.


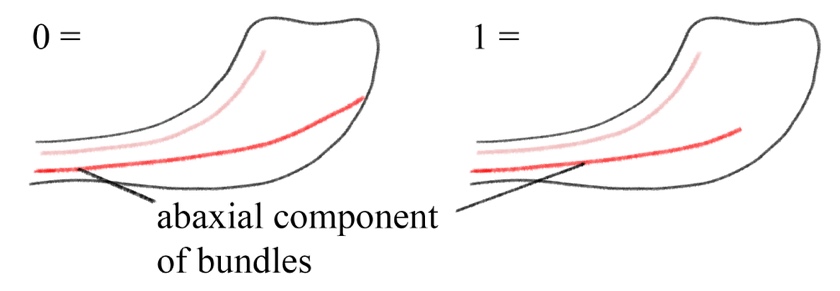


42. Abaxial component of distal ovuliferous complex vasculature forms:

0 = abaxially convex shape; 1 = abaxially concave shape; 2 = linear horizontal file; 3 = ring [*Stockeystrobus*]


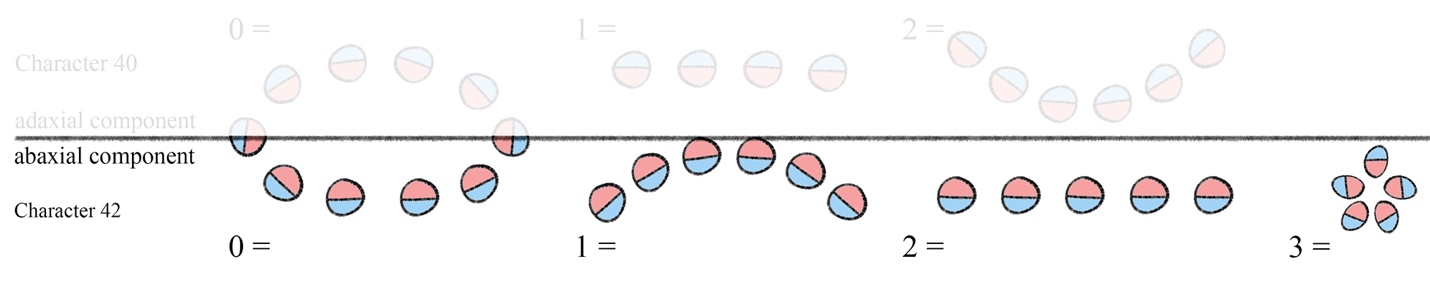


43. Abaxial component of distal ovuliferous complex vasculature with adaxially pointing notch

0 = absent; 1 = present


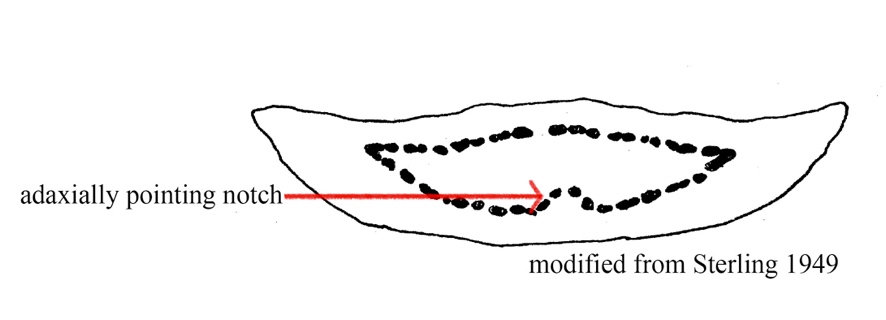


44. Vascular bundle symmetry

0 = all bundles in ovuliferous complex have bilateral symmetry; 1 = some of the bundles have ‘radial’ symmetry (“spiderweb bundles”)

45. Transfusion tissue

0 = absent; 1 = present

46. Base of ovuliferous complex supplied by resin canal(s)

0 = absent; 1 = present

The presence or absence of a resin canal is scored at (or directly adjacent to) the direct point to attachment of the ovuliferous complex to the cone axis. A very prominent resin canal is present at the point of divergence of the ovuliferous scale in some taxa like *Taxodium*, *Cryptomeria* and *Cunninghamia* (= present). A large resin canal is not present at the base of the ovuliferous in taxa like *Athrotaxis* (= absent), although smaller resin are present not far from the point of divergence.

47. Resin canals that run all the length of the ovuliferous complex uninterrupted

0 = absent; 1 = present

48. Interfascicular resin canals in adaxial component of ovuliferous complex vasculature

0 = absent; 1 = present

49. Interfascicular resin canals in abaxial component of ovuliferous complex vasculature

0 = absent; 1 = present

50. Ovule orientation before pollination

0 = erect; 1 = inverted

51. Ovule orientation after pollination

0 = erect; 1 = inverted

52. Number of rows of seeds

1 = one; 2 = more than one

53. Seed shape

0 = flat; 1 = not flat

54. Winged seeds

0 = absent; 1 = present

55. Seed scars on ovuliferous complex

0 = conspicuous; 1 = inconspicuous

56. Seed position on mature ovuliferous complex

0 = attached on basal half; 1 = attached on distal half
